# *In silico* model of the human ClC-Kb chloride channel: pore mapping, biostructural pathology and drug screening

**DOI:** 10.1038/s41598-017-07794-5

**Published:** 2017-08-03

**Authors:** Maxime Louet, Sara Bitam, Naziha Bakouh, Yohan Bignon, Gabrielle Planelles, David Lagorce, Maria A. Miteva, Dominique Eladari, Jacques Teulon, Bruno O. Villoutreix

**Affiliations:** 1 INSERM, UMR_S 973, Université Paris Diderot, 39 rue Hélène Brion, 75013 Paris, France; 2 Sorbonne Universités, UPMC Université Paris 06, UMR_S 1138, Centre de Recherche des Cordeliers, F-75006 Paris, France; 3 Service d’Explorations Fonctionnelles Rénales, Hôpital Felix Guyon, CHU de la Réunion, St Denis F-97400, Ile de la Réunion, France et Inserm U1188, Diabète athérothrombose Thérapies Réunion Océan Indien (DéTROI). Université de La Réunion; CYROI, 2, rue Maxime Rivière, Sainte Clotilde, La Réunion 97490 France

## Abstract

The human ClC-Kb channel plays a key role in exporting chloride ions from the cytosol and is known to be involved in Bartter syndrome type 3 when its permeation capacity is decreased. The ClC-Kb channel has been recently proposed as a potential therapeutic target to treat hypertension. In order to gain new insights into the sequence-structure-function relationships of this channel, to investigate possible impacts of amino-acid substitutions, and to design novel inhibitors, we first built a structural model of the human ClC-Kb channel using comparative modeling strategies. We combined in silico and *in vitro* techniques to analyze amino acids involved in the chloride ion pathway as well as to rationalize the possible role of several clinically observed mutations leading to the Bartter syndrome type 3. Virtual screening and drug repositioning computations were then carried out. We identified six novel molecules, including 2 approved drugs, diflusinal and loperamide, with Kd values in the low micromolar range, that block the human ClC-Kb channel and that could be used as starting point to design novel chemical probes for this potential therapeutic target.

## Introduction

The large ClC family of chloride channels and chloride/proton exchangers was discovered by Jentsch and associates who cloned the chloride channel of the electric organ of the Torpedo ray (named as ClC-0)^1^. Several members of this family play important roles in the health and disease states via their implication in hereditary diseases, such as myopathy, osteopetrosis, Dent’s disease and Bartter syndrome^2–4^. Within the kidneys, two ClC channels, associated to the regulatory subunit Barttin, play a key role in NaCl absorption, thus participating in the control of blood pressure^5^. Specifically, the ClC-Kb chloride channel, and to a lesser extent the ClC-Ka channel, command the basolateral step of chloride absorption in the thick ascending limb, the distal convoluted tubule and the intercalated cells of the connecting tubule-collecting duct^5^. They are also necessary to regulate urine concentration^3, 6^. Bartter syndrome (BS) is an autosomal recessive disorder, characterized by renal NaCl loss, hypokalemic metabolic alkalosis, and secondary hyper-aldosteronism with normal-to-low blood pressure^5^. Among the four types of genetic variants of BS, three are linked to renal chloride channels defects. BS type 3 is due to loss of function of ClC-Kb^7, 8^, type 4a is due to mutations in the *BSND* gene encoding the protein Barttin^9^, and type 4b is a digenic disease due to loss-of-function mutations of the two genes encoding renal chloride channels, *CLCNKA* and *CLCNKB* ^10^. Mouse models devoid of ClC-K2 (ClC-Kb ortholog) or ClC-K1 (ClC-Ka ortholog) display contrasting phenotypes, ClC-K2 KO animals show hallmarks of human Bartter syndrome^11^ while ClC-K1 KO animals display diabetes insipidus^6, 12^. Nevertheless, ClC-Ka, as well as ClC-Kb polymorphisms have been associated with predisposition to hypertension^13, 14^.

Hypertension, one of the most common human diseases, is a major risk factor for cardiovascular diseases and kidney failures^15^. Genetic studies^16^ have confirmed Guyton’s pioneering hypothesis that hypertension necessarily implies excessive salt (NaCl) handling by the kidney^17^. Combination therapy in hypertension patients includes diuretics that target the thick ascending limb and the distal convoluted tubule. However, there is general agreement that it would be helpful to diversify available medication as a tool against resistant hypertension. In this context, ClC-Ka and more particularly CLC-Kb, which is present along the entire distal nephron where finely tuned NaCl absorption occurs, constitute valuable targets for the development of new drugs in the field of hypertension^18, 19^. Several key studies along this line have been performed in order to identify inhibitors for these two channels and to delineate a possible drug binding area on ClC-Ka. Yet, at present, the pharmacology of these channels remains incomplete^20–25^.

Like other ClC proteins, the ClC-Kb chloride channel is a transmembrane protein active as an homodimer^26^. Each 687 amino-acid long protomer is composed of a TM domain comprising 17 α-helices and two intracellular cystathionine beta-synthase (CBS) domains. In spite of its high biological importance, the structure and functions of ClC-Kb are still not well understood. Yet, there are several ClC-Kb homologues with known 3D structures (or part of the structures) that can be used to gain structure-function insights. The experimental structures of the eukaryotic CLC transporter CmCLC^27^, the bacterial *Escherichia coli* ClC transporter EcClC^28^, and the cytoplasmic domain of the ClC-Ka chloride channel^29^ have been reported and Gradogna *et al*.^30^ have build a structural model for ClC-Ka using as template the bacterial EcClC transporter which shares a lower sequence identity with the ClC-Kb (28% for the 17 TM α-helices) than the CmCLC channel (33% for the 17 TM α-helices). However, while the EcClC transporter can be considered an interesting template to investigate the transmembrane regions of the ClC-Kb or ClC-Ka channels, it lacks the intra-cellular cytoplasmic domain. Of importance, the structure of the bovine ClC-K was very recently determined using cryo-electron microscopy^31^ and will definitively help to gain novel knowledge about this receptor.

In the present study, we developed a structural model of the human ClC-Kb channel using all the available relevant structural 3D templates. The initial structural models were then refined using energy minimization and other simulation tools with the protein embedded into a cell membrane model. We validated the predicted structure using several structural analysis protocols and then mapped and analyzed in 3D several known and new amino acid substitutions. Such analysis, when the amino acid variation is identified in patients, has been referred to as biostructural pathology^32, 33^. We then used virtual screening strategies to search for novel compounds that could block the protein. The putative ClC-Kb binders identified in silico were tested on xenopus oocytes. From such investigations, we are able to propose new chemicals that inhibit human ClC-Kb chloride permeation.

## Results and Discussion

Although a lot of progress has been made during the last decade in the field of membrane protein crystallization, the structural investigation of such proteins remains very challenging^34^. Indeed, according to the Protein Data Bank of Transmembrane Proteins (PDBTM)^35^ of March 2017, less than 4% of all entries from the Protein Data Bank^36^ are membrane proteins. At present, there is one experimental structure of the bovine ClC-K obtained by cryo-electron microscopy co-crystallized with Fab fragments. Here, we developed a structural model of the human protein and then mapped onto the structure previously reported experimental data and new data that we have generated for this study. The structural model was then used to search for chemical compounds that should help to understand further the function of the ClC-Kb channel.

### Model validation

**Figure 1:**
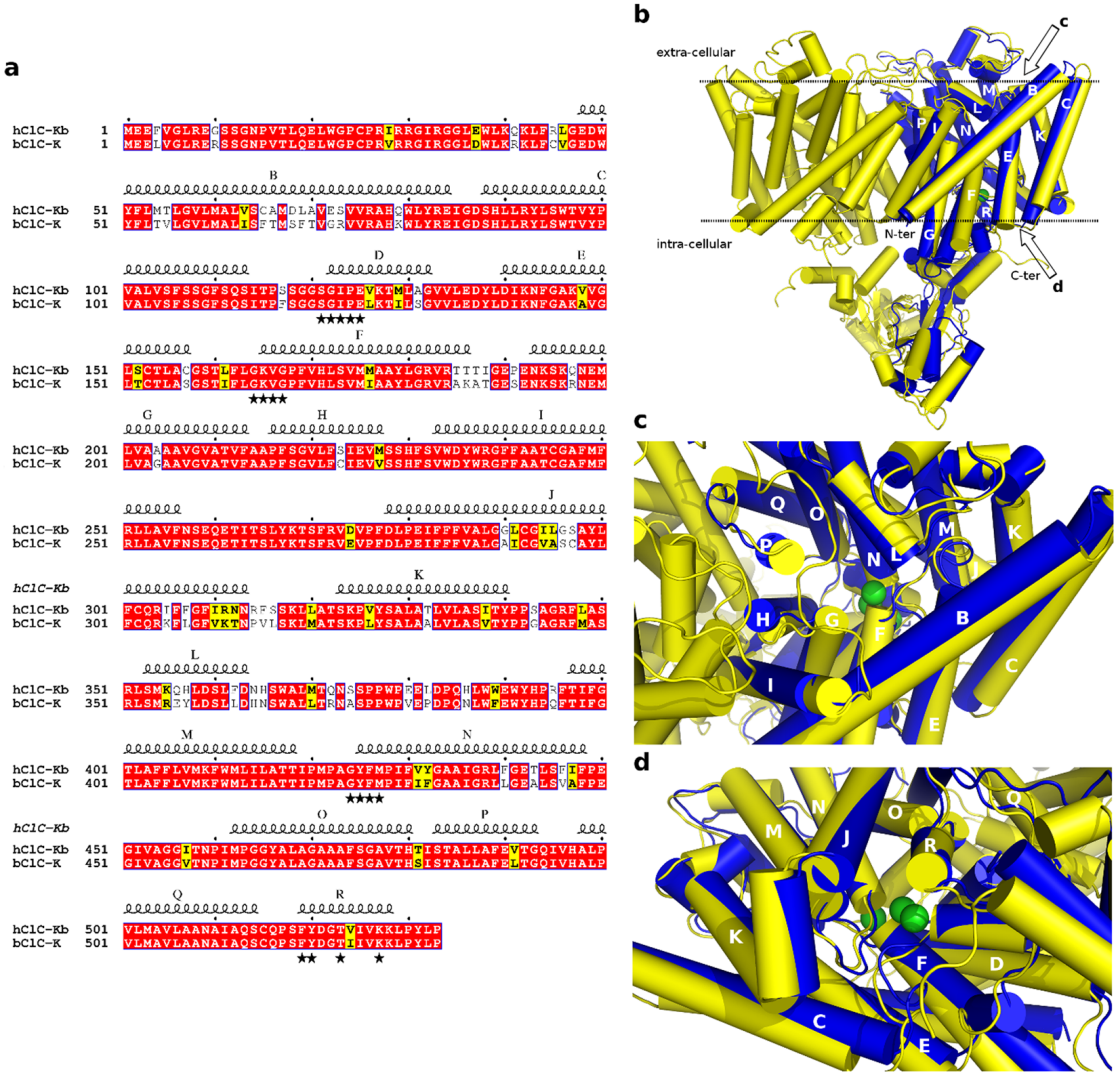
Sequence and structural alignment between the bovine ClC-K (template) and the human ClC-Kb (model). (**a**) Sequence alignment of the human ClC-Kb (hClC-Kb) and the bovine ClC-K (bClC-K) used for the human ClC-Kb model generation. The program ESPript was used to display the alignment^89^. Secondary structures are presented on top of the sequences, conserved residues are boxed and within these regions, residues that are considered similar are highlighted in yellow. (**b**–**d**) Structural alignment between the known bovine ClC-K channel structure (blue) and the equilibrated model of the human ClC-Kb (yellow). The proteins are depicted in cartoon and the known position for the chloride ions (PDB structures 1OTS and 3ORG) are represented as green spheres. The helices are labelled according to the standard nomenclature. The b panel represents a side view of the channel (the dashed line being the membrane) while the c and the d panels represent respectively an extracellular view and an intracellular view.

We used the bovine ClC-K structure (PDB id 5TQQ – better resolution than 5TR1^31^) to build the main part of the human ClC-Kb model but also considered two related structures, the eukaryotic CmCLC channel (PDB id: 3ORG^27^) and the bacterial EcClC transporter (PDB id: 1OTS^28^). One challenging part in the modelling of transmembrane proteins is generally due to the relatively low-sequence identity between the query and the experimental structural templates. However, proteins embedded into biological membranes possess a very similar fold for the core regions even with a sequence identity of about 20%^37^ and here, as the sequence identity between the bovine and the human species is high (about 84%), it is clear that the resulting human 3D model should be accurate. Yet, it was not possible at this stage to build accurately the N-terminal part (including the first helix) of the protein. In the following, all the channel helices are named alphabetically from A to R, according to the proposed nomenclature of Dutzler *et al*.^38^. Loops connecting helices are named in reference to the α-helices found before and after each loop, as an example, the I-J loop connects the I and J helices. The sequence alignment between the TM domains of the human ClC-Kb and the bovine ClC-K channels is depicted in Fig. 1. In this family of proteins, the helices are almost strictly conserved, except the helices B, J and L that are less conserved. Helix L is found in contact with the solvent and might interact with extracellular partners. Interestingly, helices B and J are known to interact with Barttin, a protein that regulates the targeting to the membrane and the ClC-K gating^39–41^.

The global fold and the pore of the final ClC-Kb model (colored yellow) are shown in Fig. 1 together with the structure of the bovine ClC-K channel (blue). The physical reliability of the ClC-Kb model, before and after refinement, has been assessed using the MolProbity server^42^. Validation statistics are shown in the Supplementary Figs S1, S2, S3, S4, the Supplementary Table S1 and the Supplementary Text S1. We then located the charged residues in the structure of the human model and in the experimental bovine structure as conservation of charges (e.g., salt bridges) can be important for both, evaluation of the model quality and to understand the function of a protein. As compared to the bovine experimental structure, we observed one additional salt-bridge in the human model while the other salt-bridges are conserved. The additional salt-bridge in the human protein involves residues D68 and K165. It is noteworthy that Picollo *et al*. noticed that amino acid 68 determined partly ClC-K sensitivity to blockers^43^ and it should be mentioned that K165 is part of the pore (see below). We also observed small differences in the electrostatic potential between the human and the bovine proteins, mainly at the dimer interface (Supplementary Fig. S5). However, we believe that these differences are mainly due to side chain rearrangements and equilibration after the MD simulation performed on the human model. Overall, the high sequence identity and the different investigations reported here and in the supplementary material all suggest that the human ClC-Kb homology model embedded into a POPC membrane is of high quality and suitable for further structural analyses.

### How a chloride ion could travel into the ClC-Kb pore?

**Figure 2:**
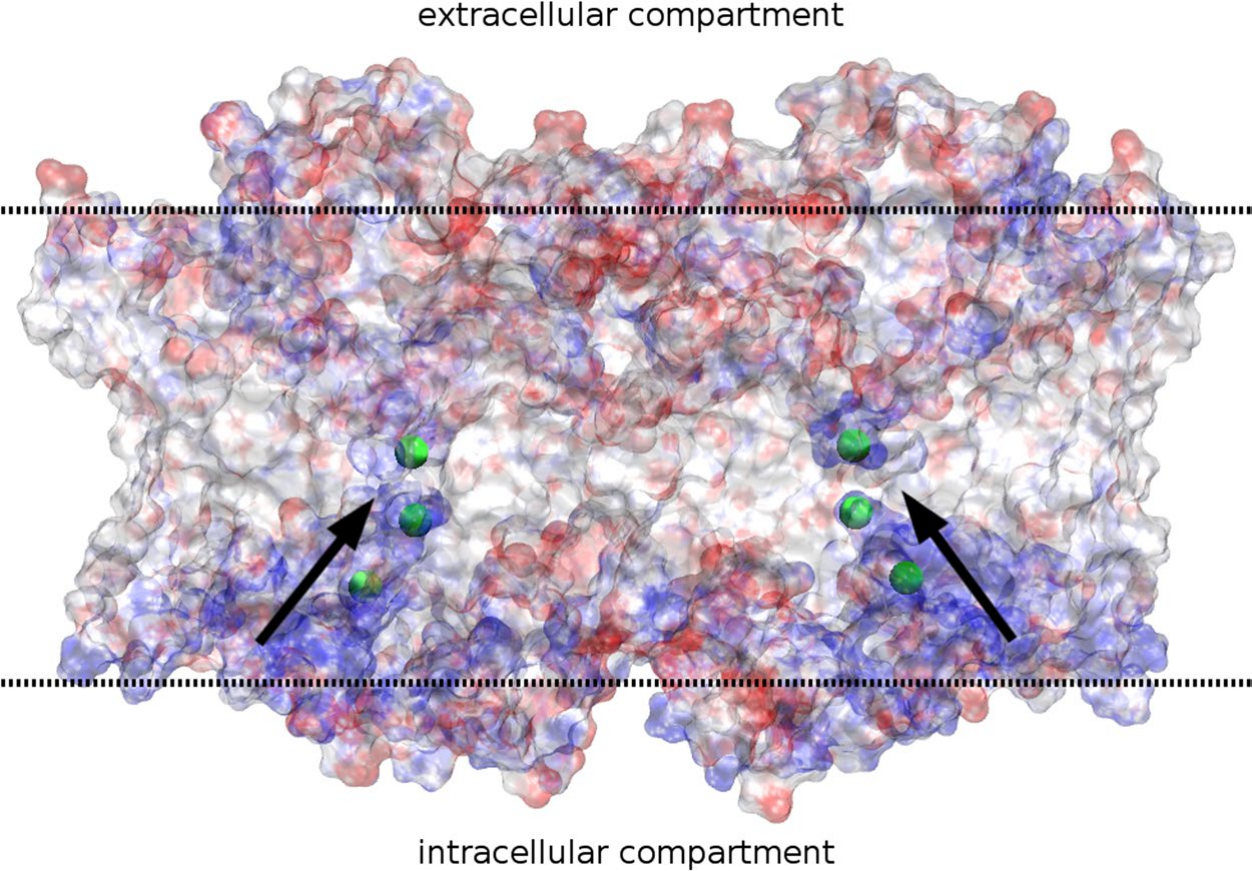
Electrostatic potentials of the TM part of the human ClC-Kb channel. View of the channel through the longitudinal axis of the membrane represented by dashed lines. The electrostatic potential calculated for the full system (protein and membrane) is mapped onto the protein surface from −5 (red) to +5 (blue) kcal.mol^−1^. Arrows represents the ion path from the intracellular compartment to the ion gate. A transparent surface representation is used in order to show the channel interior.

**Figure 3:**
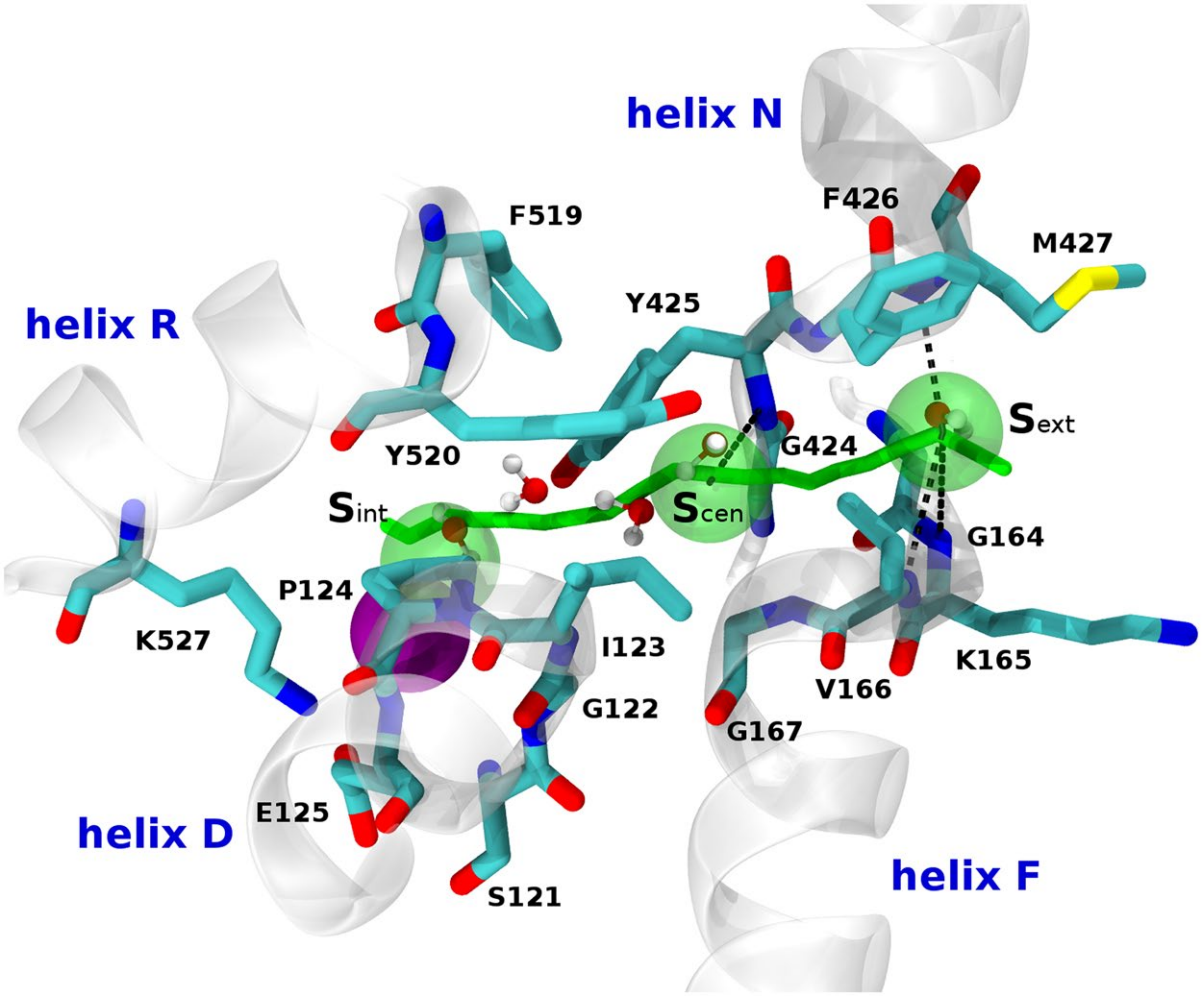
Chloride path into the ClC-Kb pore. The three experimentally known positions of the chloride ion (S_ext_, S_cen_ and S_int_) in CmCLC and EcClC are shown (green spheres). The predicted path of the chloride ion obtained from the MD simulation is shown as a green stick. Important helices are shown in a cartoon representation. Residues forming the channel pore are represented as sticks and the black dotted lines represent putative electrostatic interactions between the three possible chloride ion positions and the NH group of the protein backbone. The chloride ion interacting with the pore during the MD simulation is represented as a purple sphere. The water molecules present in the simulation are in a ball and stick representation.

ClC-Kb and ClC-Ka are not Cl-/H + exchangers as CmCLC and EcClC but chloride channels^44–48^. A proposed molecular mechanism emphasized the central role of the glutamic acid at position 210 (position 166 in ClC-Kb) for the Cl- gating and the H + exchange in CmCLC. However, this gating model is not valid for the ClC-K family. We investigated the possible structural mechanisms involved in the chloride ion pathway using the ClC-Kb model structure (Figs 2 and 3). We initially assumed that the ClC-Kb pore could be determined by homology with the pores of the bovine ClC-K templates and the CmCLC and EcClC structures where chloride ions have been co-crystallized with the proteins.

First, in order to determine if electrostatic forces could guide the ion into the pore, we investigated the electrostatic potentials of the most representative conformer of the full ClC-Kb protein explored during our simulation. The Adaptive Poisson Boltzmann Solver was used and the results were mapped onto the protein surface (Fig. 2). The electrostatic potential of the inner pore and the intracellular side of the pore were electropositive. This property should contribute to the attraction of the negative chloride ions to and along the pore. After structural inspection of the channel, it seems that this physico-chemical character is in part due to the NH-backbone groups of residues S121, G122, I123, E125, K165, V166, F426 and M427 and the K527 side chain. Indeed, the free NH groups of these residues face the inner part of the pore and coat the surface with electron donors. Interestingly, in the region corresponding to the C-terminal parts of the helices J and R (intracellular, close to the ion pore), we observed three imbalanced positively charged residues, R311, R314 and K528, that increase the electrostatic potential seen in this region. Therefore, these residues could attract negative ions toward the pore entrance. This charge imbalance was also present in the template but to a lesser extent (human R311 is a lysine in the bovine channel while human R314 is a proline).

Second, to keep track of the pore plasticity and shape, and to analyze each chloride site, we took advantage of the structural information extracted from the MD simulation. The five most representative conformations of the pore explored during the MD simulation were used to determine the possible ion pathways and the key residues involved in ion permeation. The ion pathway observed experimentally (based on CmCLC and EcClC structures) could be retrieved in the model by the Caver software^49^ (Fig. 3). This strongly suggests that the pore shape is essentially conserved between ClC-Kb and other CLC channels. A visual inspection of the same five most representative structures of the ClC-Kb pore provided some additional insights into the amino acid structures and dynamics of this system. The protein being solvated with water and Na+/Cl- ions in our simulation, we observed a full solvation of the pore. Very interestingly, the solvent molecules (very similar in size to a chloride ion) filled the pore and moved to positions identical to the experimentally-known position of the chloride ion S_ext_, S_cen_ and S_int_^27^ as well as to two other intermediate positions in between S_cen_ and S_int_, in the vicinity of helix D (Fig. 3). In addition, we observed the interaction between a free chloride ion present in the solution of our simulated system and the K527. The interaction was very stable during the simulation after the first two nanoseconds.

**Figure 4:**
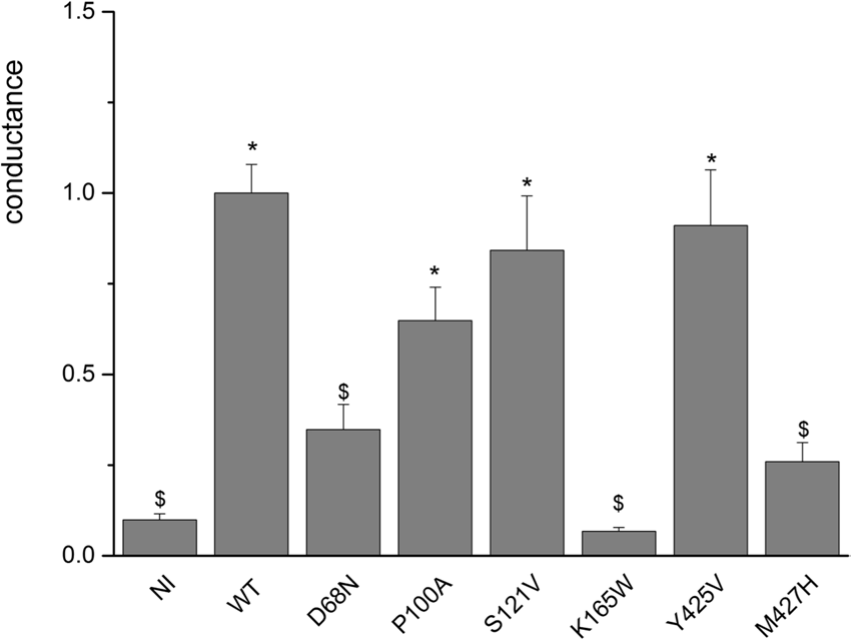
Conductance of ClC-Kb mutants. Conductance at + 60 mV in solution containing 20 mM Ca^2+^ at pH 9.0 is normalized to the mean value for wild-type (WT) ClC-Kb. Each column represents the mean ± SEM for the conductance (NI, n = 22; WT, n = 31; D68N, n = 18, P100A, n = 13, S121V, n = 13, K165W, n = 9; Y425V, n = 6; M427H, n = 9). *P < 0.05 is the difference between WT or mutant ClC-Kb *vs* NI. ^$^P < 0.05 is the difference between NI or mutant ClC-Kb *vs* WT ClC-Kb.

The visual inspection informed us about the importance of helices F and N N-terminus (K165, V166, F426 and M427) in between the S_ext_ and the S_cen_ chloride position in the pore (Fig. 3). These structural elements should have a predominant role in the chloride pathway. In fact, we observed the opening of the pore at this specific position during the MD simulation. The opening was mainly the consequence of a rotation of the V166 side-chain. In addition, we suggest that the helix F could bend slightly and expose its H-bond-free NH groups following the structural analysis of the CmCLC and the EcClC experimental structures (see Supplementary Figure S6). We propose that this tilt could facilitate the transition for the release (or binding) of the ion at the S_ext_ position. We suspect the conserved proline in the ClC-K family, at position 100 of ClC-Kb (within the helix C), to play a role in this motion. The proline breaks the helix C N-terminus and bends the whole helix B C-terminus region. As a consequence, helix B could undergo a spreading motion from the rest of the channel as seen in during the equilibration step of the simulation. Helix spreading might act on D68 (present on helix B), a residue that interacts directly with K165 (helix F, N-terminus segment). To test the importance of these residues and the reliability of these observations, we generated three protein mutants (D68N, P100A and K165W) that were functionally tested in the Xenopus oocyte system (Fig. 4). K165W corresponds to the wild-type residue in CmCLC and D68N to the wild-type residue in ClC-Ka. The current carried by the D68N and K165W mutants was not different from the one measured in non-injected oocytes, confirming their structural and functional role in the chloride pathway. Further, these two mutant proteins were expressed at the membrane at the same level as the wild-type (data not shown). We observed a non-significant 30%-decrease in P100A current compatible with a possible involvement of this amino acid in the shaping of the ion pathway. In addition, our model locates the M427 side chain at the exit/entrance of the channel pore, suggesting that this residue might participate in the external positioning of the chloride ion. We generated a M427H variant to evaluate this hypothesis. The histidine side chain is more rigid than the long flexible methionine side chain. Such hydrophilic residue at this position, in contact with the solvent, should not induce structural problems. In homologue sequences, at this position, we observe essentially smaller than histidine (i.e. leucine, valine, alanine). Very interestingly, we observed no current for this mutant (Fig. 4) that was however expressed at the membrane as the wild-type protein. We propose that M427 might be very important for the ion pathway, even if the amino acid conservation at this specific position is low.

The intermediate and central positions of the chloride ion, called S_int_ involve I123, Y425 and F426. I123 and E125 structure the pore mainly via their backbone atoms, whereas the side chain of Y425 could act on the central position of the chloride ion. However, no current decrease (as compared to WT) was observed when testing the Y425V mutant in the oocyte expression system (Fig. 4), indicating that the tyrosine side chain should be very stable and does not lock access to the pore. Yet, we propose that helix D might have a closing role in the ClC-Kb pore. Indeed, this helix could slightly shift along its axis and this might be sufficient to block the central position for the chloride ion as well as the intermediate positions. This suggestion results from the analysis of the movement of some water molecules during the simulation.

The intracellular position of the chloride ion, called S_int_, could be stabilized by interactions with the NH atoms of the helix D N-terminal segment and possibly by the K527 side chain. The S121 side-chain is known in EcClC to have a predominant role in chloride pathway^4^ but this does not seem to be the case in the bovine X-ray template and in the human protein. To test this hypothesis, we substituted serine 121 by a larger and hydrophobic valine residue, and this change did not disturb the ion pathway, since the current of S121V was similar to that of the wild-type protein (Fig. 4).

Overall, beside the possible opening/closing of the S_ext_ position by the helix F-terminus bending, and the rotation of the valine 166 we did not observe other possible gating for the ClC-Kb channel.

### Structural analysis of previously reported ClC-Kb amino acid substitutions

Forty three apparently detrimental point mutations in the TM region (excluding the linker between the TM domain and the CBS domain) of the ClC-Kb channel, leading to Bartter syndrome 3, have been reported in human^5^ but only a few have been functionally analyzed. Some of these experimental results are reported in Table 1.

**Table 1:** ClC-Kb mutations involved in the Bartter syndrome type 3 with available experimental data.

Variant	Protein region (residue distance to the pore)	Experimental Remaining current	Polyphen2.0 Probability of Damage	PoPMuSIC ΔΔG (kcal.mol^−1^)	FoldX/ENCoM ΔΔG (kcal.mol^−1^)	Exposition to solvent	ConSurf Scale	Interactive structural analysis of the 3D model
G246R	α helix I (~10 Å)	None	Probably damaging	0.89	2.35	Buried	8	Steric clashes, charged residue in the protein core, possible disruption of the dimer
A349D	K**-**L loop (~4 Å)	None	Probably damaging	0.84	2.48	Buried	8	Charged residue in the protein core and clashes with surrounding side chains
R438C	α helix N (~14 Å)	None	Probably damaging	0.99	0.96	Buried	7	Loss of a salt-bridge with E442
R438H	α helix N (~14 Å)	None	Probably damaging	0.30	0.32	Buried	7	Steric clashes in a packed environment
L439P	α helix N (~15 Å)	None	Probably damaging	2.95	2.52	Exposed to the membrane	7	Introduce a proline inside an α-helix
G424E	M-N loop (~0 Å)	None	Probably damaging	2.07	2.84	Buried	9	Serious clashes in a packed environment, charged residue in the protein core (part of the pore)
A204T	α helix G (~9 Å)	0 to 25%	Probably damaging	0.86	0.84	Buried	6	Steric clashes, could push away the α helix G from the α helix F (part of the pore)
P124L	α helix D (~0 Å)	0 to 73%	Probably damaging	0.01	−0.13	Partially exposed	7	Some steric clashes, perturbs the E125-K527 salt bridge (part of the pore), removal of a Pro at the beginning of the helix
T115P	α helix C (~8 Å)	18%	Probably damaging	0.90	1.28	Exposed	5	Add a proline at the end of an α-helix
Y432H	α helix N (~4 Å)	20%	Possibly damaging	1.86	1.33	Exposed to the membrane	4	Charged residue in contact with the membrane lipids
L139P	D-E loop (~6 Å)	27%	Probably damaging	2.35	1.13	Partially exposed	7	Destabilization of the D-E loop and disruption of the E136-R182 salt-bridge
L81P	α helix B (~21 Å)	35%	Probably damaging	1.99	1.87	Exposed to the membrane	5	Proline inside the α-helix
A210V	α helix G (~7 Å)	50%	Probably damaging	1.01	0.47	Buried	7	Some steric clashes in a relatively packed environment. Moderate damage of the protein function
G120R	C-D loop (~1 Å)	60%	Probably damaging	0.67	0,05	Exposed	8	Disruption of the E125-R527 salt bridge (nearby the pore). Partially tolerated
V170M	α helix F (~3 Å)	60%	Probably damaging	0.91	−0.56	Buried	9	Minor steric clashes, pushes away helices involved in the pore
R351W	K**-**L loop (~4 Å)	60%	Probably damaging	0.21	0.65	Exposed	3	Loss of a salt bridge with E390 but the change could be tolerated
R351P	K**-**L loop (~4 Å)	63%	Probably damaging	1.44	1.95	Exposed	3	Loss of a salt-bridge with E390 and destabilization of the L-M loop
R92W	α helix C (~20 Å)	67%	Probably damaging	0.41	0.51	Exposed	5	Modification of electrostatic interactions in the B-C loop

The mutations were sorted according to their current decrease. The investigated residues were: L81P^90^, R92W^90^, T115P^56^, G120R^56^, P124L^11, 81, 82^, L139P^56^, V170M^56^, A204T^11, 81, 82^, A210V^91^, G246R^90^, A349D^81, 82^, R351P^90^, R351W^91^, G424E^56^, Y432H^92^, R438C^90^, R438H^90^ and L439P^90^.

We investigated the possible effects of the amino acid substitutions on ClC-Kb using four different in silico tools that essentially used sequence and/or 3D structure information: Polyphen2^50^, PoPMuSIC 3.1^51^ and a combination of FoldX 3.0^52^ and ENCoM^53^. While these in silico approaches are very valuable, it is always beneficial to investigate the possible impacts of amino acid changes interactively using experimental or homology-based 3D structure such as to gain additional knowledge. Thus we also performed an interactive structural analysis for each mutation and the key features are reported in Table 1. Polyphen2 results are qualitative and classify mutations in three different groups: probably damaging, possibly damaging and benign. The tools based on the protein structure return quantitative results about the Gibbs free energy difference (∆∆G in kcal.mol^−1^) between the wild-type and protein variant. A ∆∆G of about 1 kcal.mol^−1^ can in general be considered as mildly destabilizing while a ∆∆G of about 3 kcal.mol^−1^ (and above) is considered (very) destabilizing^54^. Qualitative terms reflecting the Solvent Accessible Surface Area (SASA) of the wild-type residues are also provided. The conservation score has also been computed with the program ConSurf^55^, using the sequences of the ClC family. It is known that the different computational approaches have strengths (e.g., fast) and weaknesses (e.g., the methods in general ignore the position of a membrane, the presence of a protein-protein interface or of a pore…) and that there can be differences in the prediction outputs. As such, the interactive analysis is important to further confirm or not the possible impact of the mutations.

For 12 mutants a severe current decrease (less than 50% of the wild-type remaining current) was reported experimentally (see Table 1 and the corresponding references in the legend). These mutants (shown in Supplementary Fig. S7) are: L81P, T115P, P124L, L139P, A204T, G246R, A349D, G424E, Y432H, R438C, R438H and L439P. Six other mutants were characterized by a mild current decrease, these involve R92W, G120R, V170M, A210V, R351P and R351W (Supplementary Fig. S8 and Supplementary Text S2). Overall, this set of experimentally tested mutants, distributed all along the channel sequence, are of great interest, not only to understand better the potential impact of the mutations on the structure and function of the protein, but also to help in the validation of the accuracy of the experimental and predicted structures.

We hypothesized that at least, for the 12 substitutions with severe current decrease, the amino acid change would possibly destabilize the protein and/or affect the structure (small conformational changes to major ones, while solvent exposed mutations could play a role in modifying macromolecular interactions). This overall situation could hold for the substitutions that induce less severe current decrease (see below).

Most often, at least one automatic prediction tools and definitively the interactive structural analysis support the view that severe current decrease is associated with amino acid changes that should perturb the 3D structure. For example, the mutation P124L was predicted stable by PoPMusic and FoldX/ENCoM, while the substitution was predicted probably damaging by Polyphen. A visual inspection indicated that this amino acid change creates steric clashes and could alter the backbone orientation. The substituted residue is well-conserved in the family (see Supplementary Fig. S9) and located in a packed environment. A longer side-chain at position 124 could push helix G away from helix R (two helices which are part of the intracellular part of the pore^56^) due to steric clashes and/or by disruption of the nearby E125-K527 salt bridge (see also Fig. 3). In the same way, the A204T mutation could repulse helix F, but this helix is directly involved in the ion pathway. The A349D amino acid change is another example with some differences in the output of the prediction methods but where interactive structural analysis can help clarify the possible impact of the substitution. The A349D change could clearly modify the structure of the K-L loop region. Residue A349 is buried, in a hydrophobic environment and is well conserved in the sequences (Supplementary Fig. S9). Moreover, it has been proposed previously that a putative chloride binding site could be located around this location^57^, and thus adding a negative charge at this position could have a negative impact. PoPMuSIC output suggests this mutation to be slightly destabilizing while the two other tools predicted a relatively large destabilization effect, in agreement with the interactive analysis. Other examples of substitutions that were predicted damaging/destabilizing by all the tools used and consistent with the interactive structural analysis involve L81P, T115P, L139P and L439P. Addition of a proline in these regions of the protein should have important impacts on the local folding, orientation/packing of the helices and stability, and/or channel function, often due to drastic modification of the Phi-Psi angles. Modifications in these regions could also lead to structural modifications of the helix B, known to interact with the barttin protein, required for protein surface expression and function^39–41^ (L81P), disruption of the channel entrance (L439P), or disruption of a salt-bridge (E136-R182 in the case of L139P).

More than half of the TM domain variants (25 out of 43) have not been tested experimentally (Table 2). The analysis of the structural model can thus help to predict the possible impact of these amino acid variations. For most of the variants, all methods predicted the substitutions to be damaging, whereas, for a few others, a consensus could be found after visual inspection. The analysis was applied to the following ClC-Kb mutants: A61D, A77P, A77T, G120V, V149E, G164C, P216L, S218N, G219C, A242E, A254V, G296D, S297R, Q303P, L335P, S337F, G345S, H357Q, G424R, G437C, G437R, E442G, I447T, G465R and G470E. In total, we predicted 20 out of these 25 mutations to have an impact on the channel stability, which subsequently could lead to Bartter syndrome type 3. A discussion about the predictions of the effects of these untested mutants is provided in the Supplementary Text S3. Here again, we observed some discrepancies between the automatic in silico predictive tools, a situation that has already been observed by other research groups (see for instance^58, 59^).

**Table 2:** ClC-Kb mutations possibly involved in the Bartter syndrome type 3 in the intra-membrane region essentially lacking experimental data.

Mutation	Protein region (residue distance to the pore)	Polyphen2.0 Probability of Damage	PoPMuSIC3 ΔΔG (kcal.mol^−1^)	FoldX3/ENCoM ΔΔG (kcal.mol^−1^)	Exposition to solvent	ConSurf Scale	Interactive structural analysis of the 3D model
A61D	α helix B (~7 Å)	Probably damaging	1.64	2.35	Buried	8	Destabilization of a hydrophobic cluster in a packed environment
A77P	α helix B (~16 Å)	Probably damaging	1.90	0.71	Membrane exposed	6	Proline inside an α-helix
A77T	α helix B (~16 Å)	Probably damaging	0.96	0.00	Membrane exposed	6	—
G120V	C-D loop (~1 Å)	Probably damaging	0.52	1.60	Exposed	8	Possible disruption of E125-R527 salt bridge (nearby the pore)
V149E	α helix E (~10 Å)	Possibly damaging	0.05	−0.22	Membrane exposed	7	Charged residue within the lipid bilayer
G164C	E**-**F loop (~0 Å)	Probably damaging	−0.75	0.04	Partially solvent exposed	9	Solvent exposition of a free cysteine (part of the pore)
P216L	α helix H (~12 Å)	Probably damaging	0.80	0.42	Buried	9	Loss of a structurally important proline which breaks the α helix G
S218N	α helix H (~7 Å)	Benign	1.25	1.10	Buried	8	Steric clashes in a hydrophobic environment
G219C	α helix H (~8 Å)	Probably damaging	0.21	0.14	Buried	8	Steric clashes in a packed environment
A242E	α helix I (~13 Å)	Probably damaging	2.14	2.06	Buried	6	Large and negatively charged residue buried
A254V	α helix I (~13 Å)	Possibly damaging	1.49	0.86	Partially solvent exposed	6	Destabilization of a possible calcium ion binding site
G296D	α helix J (~7 Å)	Benign	1.85	3.09	Buried	7	Steric clashes and charged residue in the hydrophobic core
S297R	α helix J (~8 Å)	Benign	0.52	−0.90	Membrane exposed	6	Charged residue within the lipid bilayer
Q303P	α helix J (~4 Å)	Probably damaging	2.36	2.53	Buried	6	Proline within an α-helix
L335P	α helix K (~8 Å)	Probably damaging	3.07	2.92	Buried	6	Proline within an α-helix
S337F	α helix K (~14 Å)	Probably damaging	0.89	−0.06	Membrane exposed	6	—
G345S	K**-**L loop (~9 Å)	Probably damaging	0.63	3.40	Exposed	6	Steric clashes in a packed environment
H357Q	α helix L (~10 Å)	Benign	0.73	0.52	Buried	3	Possible non-native interaction with K409 (putative chloride binding site)
G424R	M-N loop (~0 Å)	Probably damaging	0.75	4.59	Buried	9	Clashes and charged residue in the protein core (part of the pore)
G437R	α helix N (~13 Å)	Possibly damaging	0.97	8.47	Buried	9	Serious clashes in a packed environment and charged residue in the protein core
G437C	α helix N (~13 Å)	Probably damaging	−0.74	1.93	Buried	9	Clashes in a packed environment
E442G	α helix N (~19 Å)	Probably damaging	1.91	3.51	Exposed	6	Loss of interactions with R438, S366, W367 and L369
I447T	N-O loop (~26 Å)	Benign	0.07	0.74	Membrane exposed	1	—
G465R	α helix O (~14 Å)	Probably damaging	0.75	0.63	Partially solvent exposed	4	Clashes in a packed environment and charged residue in the protein core
G470E	α helix O (~9 Å)	Probably damaging	2.12	2.73	Buried	9	Clashes in a packed environment and charged residue in the protein core

### Novel ClC-Kb inhibitors

So far, only a few chemicals are known to (partially) block the ClC-Kb current such as fenamates (NFA or FFA), the p-chlorophenoxy-propionic acid (CPP) like molecules, the DIDS and benzofuran derivatives^20–24, 60^. As the ClC-Kb protein is a potential target for drug discovery and that novel chemical probes could be valuable to explore its functions, we decided to use two in silico screening approaches to identify potential ClC-Kb binders and to test experimentally the identified molecules. A structure-based screening approach that uses the 3D channel model structure to find potential binders was carried out while ligand-based screening strategies were applied using as query input a known inhibitor (named RT-93) of ClC-Kb^23^. Different in silico methods can be used to search for molecules that could mimic known binders such as 2D similarities, pharmacophore modelling, 3D shape similarities, QSAR… and the selection of the methods will depend on the amount of available data (e.g., number of compounds acting of the target) at the beginning of the project^61, 62^. The idea underlying ligand-based virtual screening was first enunciated by Johnson and Maggiora, whose Similar Property Principle states that molecules that are structurally similar are likely to have similar properties^63^. An interesting known ClC-Kb inhibitor with drug-like properties is RT-93 (*K*_*d*_ of 6.0 ± 0.9 μM^23^) while some related molecules have also been reported^24^. We used 2D similarity search and colored 3D-shape comparison approaches to identity potential ClC-Kb binders that could mimic RT-93. About 2000 molecules were analyzed interactively on the computer screen and 25 molecules were selected (according to a score value and to calculated physicochemical properties) for experimental testing.

Through a receptor-based virtual screening approach, we decided to explore via docking, a subset of the US Food Drug Administration (FDA) drug collection (i.e., molecules that could be used orally). For this in silico drug repositioning experiments, the most representative structure of the MD simulation was used and 1125 FDA compounds, gathered in the last version of the e-Drug3D database^64^, were docked in a cavity that surrounds the entrance of the chloride atom. This pocket is located on the extracellular side of the protein. Interestingly, this cavity is predicted by the Surflex probe mapping utility (protomol^65^) to be the most likely small molecule ligand binding site as compared to other accessible surface cavities present on the protein. Two docking programs were used: Surflex^65^ and Autodock Vina^66^. Putative competitive inhibitors to the chloride ion were selected on the basis of a consensus given by the best scores from the two programs, and the location of the docked compound in the extracellular cleft (e.g., low energy pose nearby the chloride S_ext_ position). After interactive structural analysis, 14 drug molecules were selected.

**Figure 5:**
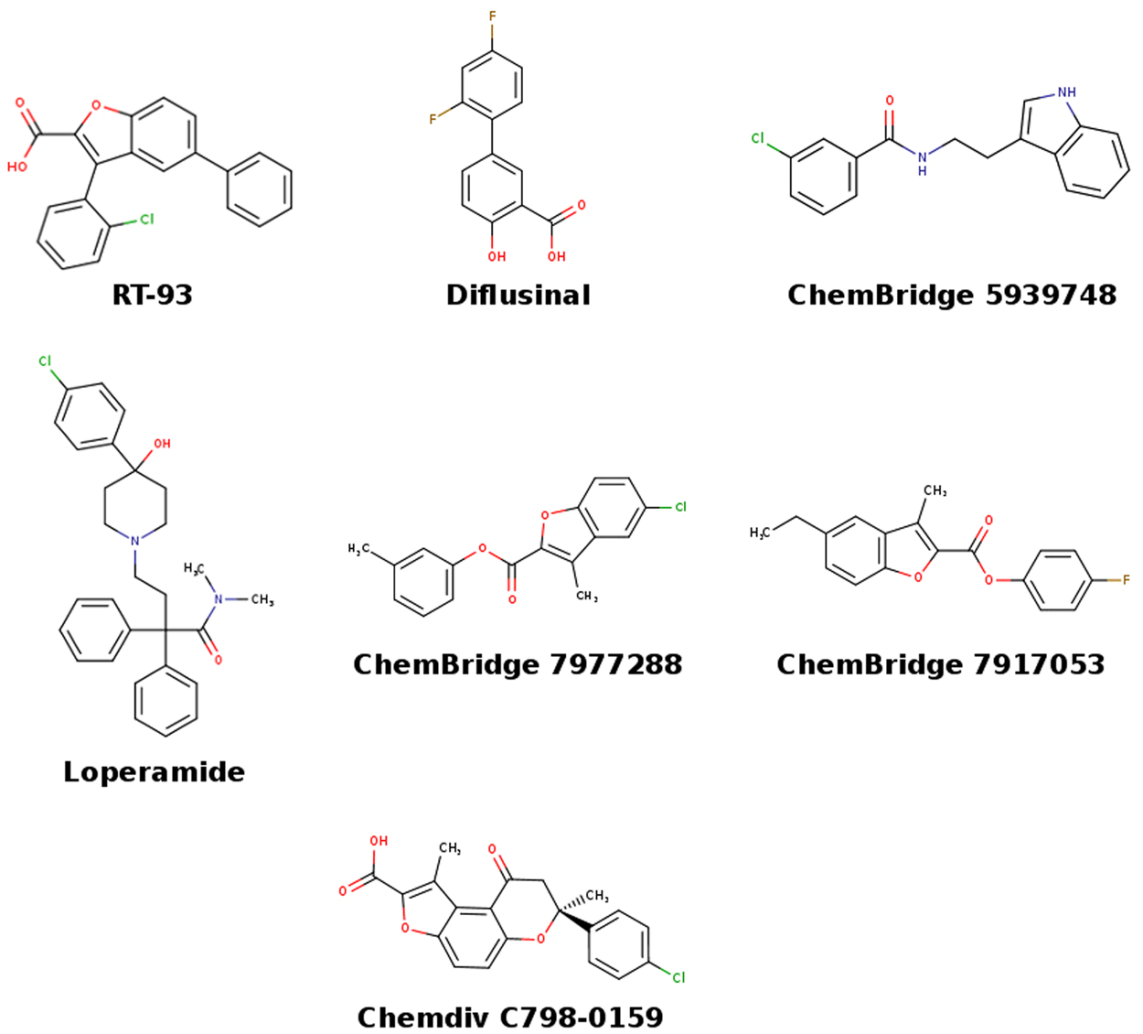
ClC-Kb inhibitors. The chemical structures of RT-93 and the other tested compounds with the highest current inhibition are shown.

We used the oocyte expression system to assess the biological effects of the 39 selected compounds. These compounds had very different inhibitory capabilities, ranging from 0% to 66% at a concentration of 100 μM (Table 3). For comparison, RT-93 (at 100 μM) decreased the current by 93%. Among the tested compounds, 6 chemically different molecules inhibited the ClC-Kb current by at least 40%: Diflunisal (non-steroidal anti-inflammatory agent), Loperamide (long-acting anti-diarrheal drug), and the ChemBridge 5939748, 7977288, 7917053, and the ChemDiv C798-0159 compounds (Fig. 5). For the top two compounds (Table 3), we determined the Kd value to be around 20 μM (diflusinal, 15.2 μM and loperamide, 33.7 μM, see Supplementary Fig. S10). This suggests that these families of chemical structures could be interesting to investigate further to design novel ClC-Kb channel inhibitors. In addition, such compounds have not been reported to interact with this protein target.

**Table 3:** Effect of tested compounds on ClC-Kb currents (expressed as the ratio of the current in presence of the compound over the current in the control).

Compound (10^−4^ M)	N	Relative current
RT93	10	0.07 ± 0.03*
Diflunisal	13	0.34 ± 0.05*
Loperamide	9	0.43 ± 0.08*
Chembridge 5939748	2	0.54 ± 0.07*
Chemdiv C798-0159	11	0.59 ± 0.05*
Chembridge 7917053	3	0.59 ± 0.09
Bupropion	9	0.62 ± 0.09*
Chembridge 6240156	3	0.66 ± 0.10
Chemdiv 3570-0493	3	0.67 ± 0.07*
Chemdiv 6228-1479	8	0.68 ± 0.08*
Chlorpheniramide maleate	5	0.75 ± 0.11
Mecloferamic acid	2	0.78 ± 0.07
Praziquante	2	0.78 ± 0.08
Chemdiv 6710-1801	5	0.78 ± 0.02*
Chemdiv 8015-5743	6	0.80 ± 0.21
Chemdiv 6228-1654	11	0.80 ± 0.22
Chemdiv 8016-1879	3	0.84 ± 0.05
Chembridge 7977288	2	0.84 ± 0.13
Chemdiv 2274-0341	7	0.85 ± 0.14
Chembridge 7917068	3	0.85 ± 0.25
Oxaprozin	6	0.87 ± 0.02*
Chemdiv 5596-0483	8	0.87 ± 0.09
Chemdiv 8018-8707	5	0.89 ± 0.02*
Chemdiv 4356-0640	8	0.89 ± 0.21
Chemdiv 4300-0449	5	0.90 ± 0.02*
Gefitinib	7	0.91 ± 0.04
Chembridge 14247123	3	0.92 ± 0.14
Chemdiv G856-6279	6	0.94 ± 0.10
Chemdiv 3570-0508	8	0.94 ± 0.04
Leflunomide	6	0.96 ± 0.02
Haloperidol	4	0.99 ± 0.06
Chemdiv 3931-2123	8	1.05 ± 0.05
Felodipine	4	1.06 ± 0.09
Baclofen	7	1.08 ± 0.08
Chemdiv 4356-0354	5	1.13 ± 0.04*
Chemdiv 4356-0657	6	1.19 ± 0.14
Chemdiv C200-7628	6	1.23 ± 0.09
Carprofen	4	1.29 ± 0.44
Chemdiv C200-7500	4	1.40 ± 0.27
Chlorphensin carbamate	3	1.73 ± 0.25

*Statistically different from control, p < 0.05.

## Conclusion

We have built a 3D model for the human ClC-Kb channel and analyzed the structure of this protein in order to gain new knowledge about its function. Because it was possible to rationalize at the structural level the impact of most amino acid variations, and as the quality control of the 3D model gave favorable scores, it is clear that the predicted 3D structure is accurate and can be used to study this important potential therapeutic target. The 3D model structure was then used to identify novel chemical binders that partially block the channel. The best compounds identified in this study could be used to design novel chemical probes such as to assess further the role of this important protein in the health and disease states.

## Materials and Methods

### Comparative modelling

Comparative modelling computations were performed using the well-established program Modeller 9v14^67, 68^. To build a comparative model, MODELLER extracts distance and/or dihedral angle restraints from the template structures, adds additional restraints implied by the covalent topology (stereochemical restraints), and predicts the structure of the model by minimizing the violations of all restraints. Modeller also applies a crude refinement routine on the built models and uses a potential energy minimizer and a short molecular dynamics simulation routine under spatial constraints to remove steric clashes and explore favorable side-chain rotamers. The cryo-electron microscopic structure of the bovine ClC-K (PDB id: 5TQQ, resolution 3.76 Å^31^) was used to build the main part of the human ClC-Kb channel and the X-ray structure of the bacterial *Escherichia coli* ClC transporter EcClC (PDB id: 1OTS, resolution 2.5 Å^28^) was used for the I-J loop not present in the bovine ClC-K template.

The ClC-Kb TM domain was modelled essentially using the bovine channel due to a high sequence identity (84% identity and 91% similarity) while the EcClC I-J loop has 29% sequence identity with the corresponding region of ClC-Kb. The alignments were performed by the program EMBOSS Needle provided by the EMBL-EBI web-server^69^. No amino-acid insertion was observed between the bovine template and the human ClC-Kb. However, some amino acids were missing in the bovine ClC-K structure. These residues, located in several loops (454 to 456, 618 to 629 and 684 to 687) were handled by the MODELLER package as their sizes were lower or equal to 12 amino-acids^70^. Both ClC-Kb protomers were modelled independently. One thousand models were created using Modeller 9v14^67, 68^. The generated models were all ranked with the Discrete Optimized Protein Energy (DOPE)^71^ method, an internal scoring function of Modeller. The best scoring model was selected and refined using Molecular Dynamics (MD) simulation carried out with NAMD^72^.

### Molecular Dynamics Simulations

In order to refine our homology models, energy minimization and MD simulations were carried out with the NAMD software^72^ using the CHARMM36 force field^73^, including the CMAP procedure to better treat the protein backbone properties^74^. Prior to any calculation, the protonation state of the amino-acids was investigated with the program DelphiPKa^75^. Accordingly, the His 230 residues of both protomers were protonated. The ClC-Kb dimer was first minimized with 2,000 steps of steepest descent followed by 5,000 steps of conjugate-gradient. The amino-acid backbone atoms were initially restrained in position with a force constant of 50 kcal.mol^−1^ with CHARMM^76^ (version 39b1). This step was necessary to remove minor steric clashes resulting from the homology modelling procedure. The energy minimized structure was then inserted in a pre-equilibrated membrane model comprising 328 1-palmitoyl-2-oleoyl- sn-glycero-3-phosphocholine (POPC) lipids. The protein position into the biological membrane was determined with the Positioning of Proteins in Membrane (PPM) web server^77^. The system was then solvated by two water shells, one of 15 Å in the z+ direction and a second of 10 Å in the z-direction. The TIP3P set of parameters was chosen to describe the water molecules^78^. One hundred and thirty-nine sodium ions and 139 chloride ions were added to keep the system neutral and to set the ionic concentration of NaCl to 0.15 M. The size of the final system comprising the protein, the membrane, the solvent and the ions was about 127 Å × 126 Å × 126 Å for a total of 212,389 atoms. The particle mesh Ewald^79^ method was used for electrostatic interactions, and the van der Waals interactions were computed with a switching function applied in the range 9–10 Å. The NPT ensemble was used (1.01325 bar and 300 K) with Langevin dynamics and a Nosé–Hoover–Langevin piston pressure control^80^. The integration step was set to 1 fs. The system was first submitted to a 2-ns MD simulation while the protein atoms were fixed in position. In a second stage, all constraints were removed, and the simulation was run for 10 ns.

### Amino acid substitutions and analysis

All amino acid substitutions (also called mutants or variants in this article) between the wild-type protein and mutant proteins observed in patients or obtained by site directed mutagenesis were investigated with different tools. We used Polyphen2^50^, PoPMuSIC 3.1^51^ and a combination of FoldX 3.0^52^ and ENCoM^53^ to predict the possible impact of the substitution on the ClC-Kb channel. Polyphen2 is mainly based on structural (known structure or homologue structures identified in databases) and evolutionary characteristics and classifies amino acid substitutions into one of the following categories: possibly damaging, probably damaging and benign according to a position-specific independent count score. The three other tools require the 3D structure of the protein and compute the Gibbs free energy difference (ΔΔG in kcal.mol^−1^) between the wild-type protein and the mutant. In practice, FoldX and PoPMuSIC use different empirical formalisms and predict the possible impact of a mutation in term of possible destabilization or stabilization of the protein under study. ENCoM predicts the effect of mutations on the protein dynamics. It has been shown that combining the ENCoM predictions with those from FoldX3.0 increase substantially the prediction accuracy. Indeed, ENCoM is more focused on the prediction of the entropy gain or loss, whereas FoldX is more focused on questions related to enthalpy^53^.

The ClC-Kb mutants or variants were generated using the psfgen package included in VMD 1.9.2^81^. The obtained structures were briefly energy minimized, atoms were harmonically constrained in position with a force constant of 0, 5, 15 and 150 for the mutated residue itself, side-chains of residues in contact with the mutated residue (distance inferior or equal to 5 Å), main-chain of the same set of contact residues and all other atoms of the protein, respectively. The Generalized Born Implicit Solvent (GBIS) was used during these computations.

### Electrostatic computations

Electrostatic analyses of the proteins were performed using the Adaptive Poisson-Boltzmann Solver (APBS)^82^. The electrostatic potential was computed using a dielectric constant of 80.0 for the solvent exposed regions and 4.0 for the buried region. The temperature was set to 300 K to fit the MD simulation parameters. The concentration of implicit sodium and chloride ions was set to 0.15 M. The atom parameters (atomic charges and van der Walls radii) for the protein and lipids were taken from the CHARMM36 force field^73^. The computed electrostatic potential was then mapped onto the protein surface calculated by the MSMS tool^83^.

### Conformational clustering

The conformations obtained along the 10 ns MD trajectory were structurally clustered using the quality threshold algorithm, included in the VMD software^81^. Three clustering protocols were used, the first one carried on the full protein; the second one on the extracellular cleft communicating with the extracellular gate of the pore; and the third one on the inner chloride pore. For the full protein, both monomers, including the CBS domain were considered except hydrogen atoms. For the extracellular cleft, residues S64, D68, V71, E72, L151, L155, A156, F162, L163, G164, K165, V166, T211, V212, F213, A214, F250, Q260, E261, T262, R351, M354, F426, M427, P428, F430, L487 and V491 main chains and side chains, excluding hydrogen atoms, have been considered. Main chains and side chains, excluding hydrogen atoms, of residues S121, I123, P124, L163, G164, K165, V166, G167, T417, G424, Y425, F426, M427 and F519 were taken into account for the clustering of the channel pore. RMSD values were used as the clustering criteria. The clustering threshold was set to 1 Å for the extracellular cleft and the inner pore whereas it was set to 1.5 Å for the whole protein homodimer. Structural centroids were then determined for each cluster.

### Predicted chloride pathway

The chloride pathway was investigated with the Caver software^49^. Only the ten most representative structures of the pore were kept for the Caver analysis that covered almost 80% of the trajectory. With regard to the Caver parameters, a probe radius of 0.8 Å, a shell radius of 3 Å and a shell depth of 4 Å were used. The starting point to search the pore was determined near the known extracellular position of the chloride ion (S_ext_). The caver results from the ten selected structures according to the process described above were then aggregated and analyzed.

### Receptor-based virtual screening

Virtual screening of a subset (oral) of the Food Drug and Administration (FDA) approved drugs gathered in the last version of the e-Drug3D database^64^ was performed. Docking calculations of 1125 compounds referenced as orally available (molecular weight around 500 Da) were carried out with Surflex 2.7^65^ and Autodock Vina^66^. The compounds were prepared in part with our online server FAF-Drugs^84^. The major protonation state at pH 7.4 was predicted for each molecule with the cxcalc major microspecies plug-in of the ChemAxon JChem 5.4.1 package (www.chemaxon.com). The 3D conformation of the compounds was generated using the Corina package^85^.

The docking search space for Surflex (protomol) can be performed using a user defined area or the package can propose the top binding pockets at the surface of a protein target. The search zone to build the protomol was chosen such as to include all residues predicted to be part of the extracellular cleft (see paragraph Conformational clustering) plus 2 Å around them. Interestingly, a blind prediction of the most likely ligand binding pockets on the channel identified this exact same position suggesting that this region can make favorable interaction with a small chemical compound. Twenty docking poses were generated and the three best-scoring poses were further considered. The Surflex option to obtain optimal pose prediction, -pgeom, was used with a RMS minimum difference cutoff of 0.85 Å between the final poses. The docking grid size for Autodock Vina was 30 × 22 × 24 Å and centered on the extracellular cleft of the channel. Ten docking poses were requested and the three best scoring poses were analyzed interactively. The exhaustiveness of the global search was set to 8. The medicinal drugs identified in silico and selected interactively as potential blockers were all tested experimentally (14 drugs in total).

### Ligand-based virtual screening

A small molecule named RT-93 has been reported to inhibit CLC-K channels^23^. This compound was used as query input and 2D similarity searches (using the PubChem 2D dictionary-based fingerprint) were performed via the PubChem online service^86^, looking for molecules with a similarity score above 80%. In addition, a 3D conformer search (PubChem3D) against the PubChem compound database was carried out over millions of chemicals available at PubChem using as input a low energy 3D structure of RT-93 generated by the Chemaxon package. The shape plus chemical feature similarity option was selected for the search^87^. The Gaussian-based similarity description and search of molecular shapes is performed with the ROCS and OEShape tools from OpenEye Scientific Software Inc, Santa Fe, NM, USA. In total, 25 molecules were selected for experimental assays.

## Experimental Methods

Human GFP-ClC-Kb and Barttin were subcloned into pTLN and pT7T3 expression vectors, respectively, for expression in *Xenopus laevis* oocytes. Capped cRNA were synthesized *in vitro* from the wild-type and mutant ClC-Kb expression vectors, and from the wild-type Barttin expression vector using the SP6 and T7 mMessage mMachine kit, respectively (Ambion, Austin, TX, USA).

Defolliculated *Xenopus leavis* oocytes were injected with 10 ng of ClC-KB cRNA and 5 ng of Barttin cRNA, and were kept at 16 °C in modified Barth’s solution containing (in mM): 88 NaCl, 1 KCl, 0.41 CaCl_2_, 0.32 Ca(NO_3_)_2_, 0.82 MgSO_4_, 10 HEPES, pH 7.4 and gentamicin (20 µg/ml). Two-electrode voltage-clamp experiments were performed at room temperature using a TURBO TEC-10CX amplifier (npi electronic, Tamm, Germany) and PClamp 9 software (Molecular Devices, Sunnyvale, CA, USA), two-three days after injection. Currents were recorded in response to a voltage protocol consisting of 20 mV steps from −140 mV to +100 mV for 800 ms from a holding potential of −30 mV. The steady-state current at the end of each voltage step was used for data analysis. The data were filtered at 500 Hz, digitized using a Digidata 1440 A analogue-to-digital converter, and Axon pClamp 9 software (Molecular Devices, Sunnyvale, CA).

For analysing the current of mutant ClC-Kb proteins, we took into account that the maximum current is obtained in the presence of 20 mM CaCl2 at pH9.0^56^. Standard solution contained (in mM): 120 NaCl, 2 KCl, 1 CaCl_2_, 1 MgCl_2_, 5 buffer; calcium-rich solution was similar except that it contained 80 NaCl and 20 CaCl_2_. These solutions were adjusted to pH 7.4 (using HEPES as a buffer) or 9.0 (using Trizma Base as a buffer). A standard solution at pH 5.0 allowed determining the zero current level. A second solution was used for the same purpose, in which 80 mM NaCl of the standard solution was substituted for NaI, since iodide at high concentration blocks ClC-Kb currents^88^. A complete current/voltage relationship was established for each oocyte under each of the experimental conditions. The chord conductance at +60 mV was calculated for each measurement as the ratio of the current at +60 mV over the difference between +60 mV and the reversal potential.

When screening the effects of drugs (10^−4^ M), we used the same solution as indicated above except that it contained 100 mM NaCl and 10 mM CaCl_2_ (adjusted at pH 7.4). Currents were first recorded in the presence of pH5.0 solution for determining zero ClC-Kb current level. The RT-93 molecule was synthesised by the Ambinter Company (www.ambinter.com); other compounds were obtained from ChemBridge (www.chembridge.com) and ChemDiv (www.chemdiv.com) while approved drugs were purchased at Prestwick (www.prestwickchemical.com). The effects of the compounds are quantified as [current in the presence of compound X- current measured at pH5.0]/[current in the absence of compound X-current measured at pH5.0].

All experimental results are shown as mean ± SEM (*n*), where *n* indicates the number of experiments. Statistical significance was analyzed by a Student’s t-test or by ANOVA followed by Holm-Sidak test using SigmaStat software (SPSS, Erkrath, Germany). *P* < 0.05 was considered significant.

## Electronic supplementary material

Supplementary data
