# Supplementary material for: In silico model of the human ClC-Kb chloride channel: pore mapping, biostructural pathology and drug screening
Source: Sci Rep. 2017 Aug 3;7:7249. doi: 10.1038/s41598-017-07794-5 (PMC5543074; doi:10.1038/s41598-017-07794-5)
Supplement: Supplementary file 1 — Supplementary data [file 41598_2017_7794_MOESM1_ESM.pdf]

In silico model of the human ClC-Kb chloride channel: pore mapping, biostructural pathology and drug screening

Maxime Louet[*a], Sara Bitam[*b], Naziha Bakouh [b], Yohan Bignon [b], Gabrielle Planelles[b], David Lagorce[a], Maria A. Miteva[a], Dominique Eladari[c], Jacques Teulon[b] and Bruno O. Villoutreix[a].

[a] INSERM, UMR_S 973, 39 rue Hélène Brion, 75013, Paris, France

[b] Sorbonne Universités, UPMC Université Paris 06, UMR_S 1138, Centre de Recherche des Cordeliers, F-75006, Paris, France

[c] Service d'Explorations Fonctionnelles Rénales, Hôpital Felix Guyon, CHU de la Réunion, St Denis F-97400, Ile de la Réunion, France et Inserm U1188, Diabète athérothrombose Thérapies Réunion Océan Indien (DéTROI). Université de La Réunion ; CYROI, 2, rue Maxime Rivière, 97490 Sainte Clotilde, La Réunion, France

*these two authors equally contributed to this publication

Correspondence to Bruno Villoutreix

bruno.villoutreix@inserm.fr

Supplementary Text S1:

The stability of the model was investigated by computing the Root Mean Square Deviation (RMSD) values (Supplementary Figure S1). The protein was very stable during the MD simulation with a RMSD of 2.5 Å for all the protein atoms (except hydrogen atoms) including long loops, which is low for a protein of this size. Then, the Cα coordinate fluctuations of the two protomers were computed and compared in order to test the reproducibility of the protopore dynamics. The Cα Root Mean Square Fluctuation values (RMSF) for each domain of both protomers are shown in the Supplementary Figure S2. The dynamical behaviour of the two protein chains are similar as the correlation coefficients between their RMSF profiles are 0.83 for the TM part (residues 48 to 531) and 0.92 for the CBS domain (residues 532 to 687). We then investigated the protein translation along the Z-axis, and the tilt angle of the protein with respect to the POPC membrane plane. These values were computed in order to test the reliability of the initial protein position into the membrane. The time evolution graphs and histograms corresponding to these two physical quantities are available in Supplementary Figure S3. The z-translation mean distance during the MD simulation, with regard to the starting position, was only 0.31 % of the mean membrane thickness (34.8 Å) and reached a maximum value of 4.4 %. The mean membrane thickness has been computed all along the MD trajectory with the VMD[1] MEMBPLUGIN[2]. The mean tilt angle of the protein TM helices observed during the simulation was about 0.7° with a maximum value at 1.7°. Either the translation or the rotation fluctuated around an equilibrium point nearly located at the initial position. Regarding these data, the protein position and rotation in respect to the membrane was stable during the 10ns MD simulation. These observations confirmed and validated the protein position obtained from the PPM web server and chosen as the starting point for the MD. Finally, the secondary structure stability has been monitored and plotted along the MD simulation (Supplementary Figure S4). The model secondary structures were very stable and we observed only very small fluctuations. For instance, only the helix E N-term of both protomers fluctuated between α-helices and 3-10 helices. Not surprisingly, some loop regions also fluctuated from random coil to turn during the simulation with no consequence on the protein stability.

Supplementary Text S2:

Residue R92, mildly conserved in the ClC family, is located in the N-terminal region of helix C and should have contacts with the phospholipids' heads, an interaction known to be important for membrane protein translocation and anchoring[3]. Although the R92W mutation was not predicted to be destabilizing (only Polyphen flags the substitution as probably damaging), we observed in the model structure the possible loss of an essentially solvent exposed and weak salt-bridge involving R92 and D87, as well as novel non-bonded interactions with the G86 and S88 backbone oxygen atoms, in the B-C linker. Substitution by a long side-chain such as methionine at position 170 (a residue that is located nearby the pore) as it is the case with the variant V170M, could lead to small clashes with I123, A205 and V226 and possible local perturbations on helices D, G and H respectively. Interestingly, this substitution was considered as stabilizing by FoldX/ENCoM while, interactively, some small clashes were visible. This change should not have dramatic effect on the function of the protein, a view consistent with the experimental data. Some experimental data for the R351P and R351W mutants suggest a decreased surface expression and decrease current. The presence of a proline or a tryptophan within the K-L loop could have an effect on the loop structure, but the most probable effects could be the loss of the salt bridge between R351 and E390 (located in the long extracellular loop (L-M loop). The interactive structural analysis agrees for the mutant R351P with the prediction tools and suggests that the R351W could be tolerated. It was important to note that these mutants have limited effects on the protein function (about 60% of the wild-type current). G120 is located in the C-D loop, close to the ion pore. The G120R was not predicted destabilizing by the structural tools used. The interactive analysis suggests that an arginine residue might not be able to adopt the phi-psi angles of the small glycine residue and could modify the loop conformation as well as the interaction between E125 and K527. Overall the analysis is in agreement with the mild current decrease and suggested a limited effect on the protein function. The mutation A210V was predicted to be destabilizing by Polyphen and PoPMuSIC. Interactively on the computer screen, steric clashes were observed after substituting alanine by a larger valine in this tightly packed region. The A210V mutation is likely to perturb the conformation/orientation of the surrounding helices (G, H and I), suggesting that the channel could be moderately damaged, in overall agreement with the experimental data.

Supplementary Text S3:

Here, we first investigated proline residues or residues that were mutated to Pro. Proline residues are often used to break helices, this is the case for ClC-Kb helices C, H, M, N, O and R (6 out of 18). Thus, the substitution of the proline 216 by a leucine in the N-term part of helix H could modify the length of the helix and then the whole conformation of the channel. This proline is conserved in every ClC family members indicated by the high conservation score further supporting its structural role. Mutations to proline within α-helices can have drastic effect on protein folding. Thus mutations Q303P and L335P (helix J and K respectively) were predicted very destabilizing by all the *in silico* tools and a visual inspection confirmed these results. This holds for the A77P substitutions.

Next, we looked at glycine residues. The conserved glycine, at position 120, close to the ion pore, was predicted destabilizing by Polyphen and the FoldX/ENCoM combination. The mutant G120V might destabilize the salt-bridge between E125 and R527 that shape the pore and should have a mild effect on the protein function. Position 164 (G164C) is very important for the channel pore and is directly involved in its extracellular part. The glycine is conserved in every homologue sequences to ClC-Kb. In addition, this mutation would expose a free cysteine to the solvent, which could react to form a disulfide bond. The same trend is observed for mutant G424R. The nature of the pore gate (constituted by the N-term parts of helices J and N) does not allow any amino acid but a glycine in position 164 and 424. Similarly, the G437 possesses surely an important structural role as it is found strictly conserved in the ClC family and the packing in this region, mainly due to the presence of L288, F440 and Y466, does not allow any other amino acid than a glycine. Interestingly, those three residues are conserved in every of the homologue sequences to ClC-Kb. G296D and G470E are unlikely to be favourable as predicted by almost all 3D based in silico approaches. Indeed, all the wild-type residues are buried into the channel and substitution by a charged residue might result in strong structural modification. In addition, all of these missense mutations would lead to longer side chains which could not fit into the tight cavities and would push surrounding α-helices. G219C was not predicted destabilizing (except by Polyphen) however a simple visual analysis informed about the highly packed environment at position 219. The cysteine would clearly not fit into this cavity and would push away helix G from helix H, right at the dimer interface. Mutation G345S was found damaging by Polyphen and FoldX/ENCoM tools. After interactive analysis it seems that the serine side chain may not accommodate well at this position. The glycine at position 465 was found substituted by an arginine in several patients. The mutant was not predicted to be destabilizing by the 3D-

based tools. However, the long positively charged side-chain does not seem to fit into a tight hydrophobic cavity. The E442G substitution is discussed below as it involves another mutation.

The other mutations involve for instance A61D, S218N. Mutants A61D, S218N are unlikely to be favourable as predicted by almost all 3D based in silico approaches. The residues are buried into the channel and substitution by hydrophilic residues might result in structural modification. In addition, all of these missense mutations would lead to the positioning of a longer side chain into tight cavities and would push surrounding α-helices. Prediction methods nearly failed to predict the most likely negative effect of the V149E mutation. The residue is located inside the membrane bilayer and all the surrounding residues that should be in contact with the hydrophobic part of the lipids are hydrophobic and/or aromatic. A negatively charged residue at this position should not be well tolerated. The same observation applies to the S297R substitution but in this case a positively charged residue would be buried in the cell membrane. These two cases demonstrate the need of knowing the protein environment in order to better predict the mutation effects and the importance of interactive structural analysis. Regarding the A242E, we observed that at position 242 in this protein family one tends to find small residues (eg. alanine, serine). Substitution of Ala by a Glu is not favorable and is indeed predicted to have a negative impact on the structure and function of the protein by all the approached used. The strong interaction network, found in the vicinity of residue 242, involving D49, K196 and R238, could be modified by the presence of this charged residue. Interestingly, the mutant A254V, located at the C-term of helix I, was predicted to be slightly destabilizing by FoldX/ENCoM and destabilizing by Polyphen and PoPMuSIC. Due to its location, we suspect that this mutation might have an impact on the channel function and especially in the calcium binding process, which regulates the channel activity[4,5,6]. Mutant H357Q was not predicted deleterious with the prediction tools. Interactive analysis suggests K409 to be in the vicinity of residue 357. The mutant side chain could then interact via H-bond with the lysine. Interestingly, K409 was proposed previously to have a role in chloride binding[4]. The mutation of the arginine residue at position 438 (R438C and R438H) has been tested experimentally and no current can be detected for such substitution. Non-bonded interactions with E442 were found to be stable along the MD simulation. The substitution E442G is expected to modify the channel structure and/or function. Interestingly, it seems that this residue and its interactions with the surrounding are crucial for the protein stability and its addressing to the plasmic membrane. In addition, E442 was found to interact with the backbone nitrogens of S366, W367 and L369 and participates to the structure of the

long extracellular loop L-M. The prediction tools supported these observations.

The effect of 3 mutations could not be rationalized in 3D at present. No consensus could be found for the following mutants: A77T and S337F, both in contact with the hydrophobic part of the lipid, these changes could thus be tolerated. Finally, the I447T appears to be tolerated in the 3D model structure. We could not reject the hypothesis that these residues are important for interaction with protein partners that regulate the ClC-Kb function.

1 Humphrey, W., Dalke, A. & Schulten, K. VMD: visual molecular dynamics. *Journal of Molecular Graphics* **14**, 33-38, 27-28 (1996).

2 Guixà-González, R. *et al.* MEMBPLUGIN: studying membrane complexity in VMD. *Bioinformatics (Oxford, England)* **30**, 1478-1480 (2014).

3 Schiffer, M., Chang, C. H. & Stevens, F. J. The functions of tryptophan residues in membrane proteins. *Protein Eng* **5**, 213-214 (1992).

4 Faraldo-Gomez, J. D. & Roux, B. Electrostatics of ion stabilization in a ClC chloride channel homologue from Escherichia coli. *J Mol Biol* **339**, 981-1000 (2004).

5 Gradogna, A., Fenollar-Ferrer, C., Forrest, L. R. & Pusch, M. Dissecting a regulatory calcium-binding site of CLC-K kidney chloride channels. *The Journal of General Physiology* **140**, 681-696 (2012).

6 Gradogna, A. & Pusch, M. Molecular Pharmacology of Kidney and Inner Ear CLC-K Chloride Channels. *Frontiers in Pharmacology* **1**, 130 (2010).

Supplementary Table S1: Validation statistics for both models, before and after structure refinement, using the MolProbity server.

	PDB template 5TQQ	Model without refinement (Modeller 9v14 only)	Model with refinement (Modeller 9v14 + energy minimization + MD)
Clashscore[a] (lower the better)	6.22	2.62	1.31
Poor rotamers (goal < 0.3%)	0.89 %	2.72 %	2.63 %
Favoured rotamers (goal > 98%)	93.60 %	90.34 %	91.56 %
Ramachandran outliers (goal < 0.05 %)	0.12 %	1.25%	1.65 %
Ramachandran favoured (goal > 98%)	95.18 %	93.57 %	91.46 %
MolProbity score[b] (lower the better)	1.68	1.80	1.68
Cβ deviations >0.25Å (goal 0 %)	2.10	11.68 %	1.85 %
Bad bonds (goal 0%)	0.00 %	2.57 %	0.00%
Bad angles (goal 0.1%)	0.05 %	4.01 %	0.64 %

[a]Clashscore is the number of serious steric overlaps (> 0.4 Å) per 1000 atoms.
[b]MolProbity score combines the clash score, rotamer, and Ramachandran evaluations into a single score, normalized to be on the same scale as the X-ray resolution.

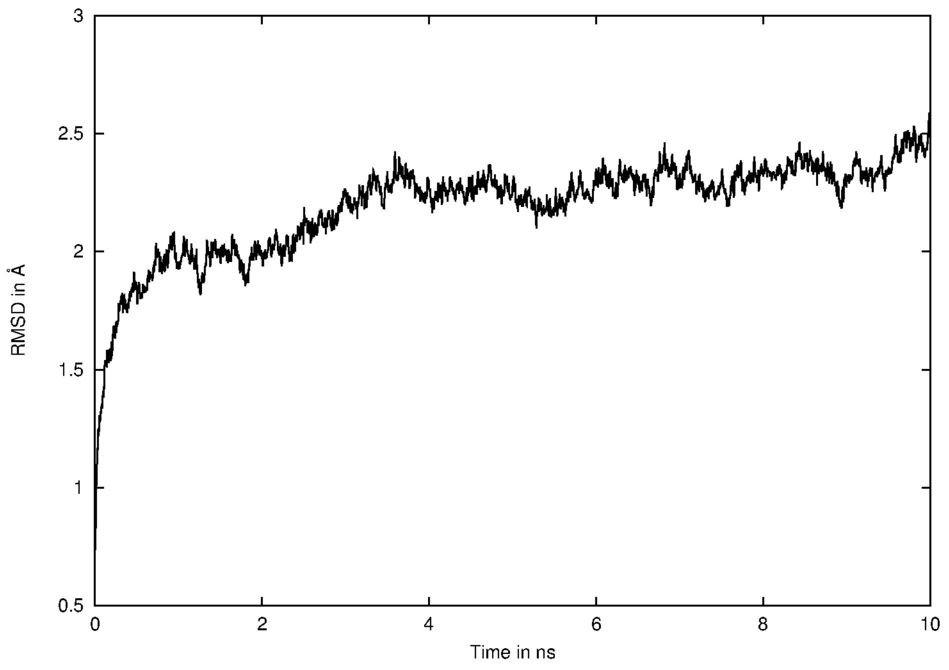

Supplementary Figure S1: RMSD of all heavy atoms of ClC-Kb along the 10 ns MD simulation.

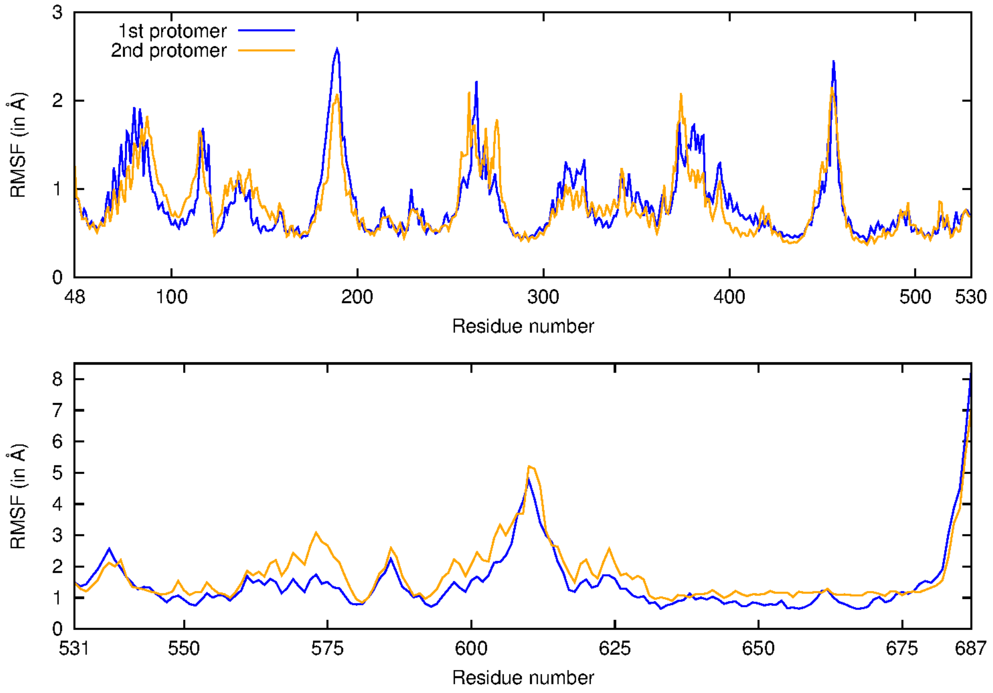

Supplementary Figure S2: Cα Root Mean Square Fluctuation of the TM part (top) and the CBS domain (bottom) of the first (blue) and the second (orange) ClC-Kb protomers. The correlation coefficients between the two protomers were 0.83 and 0.92 for the TM part and the CBS domain respectively.

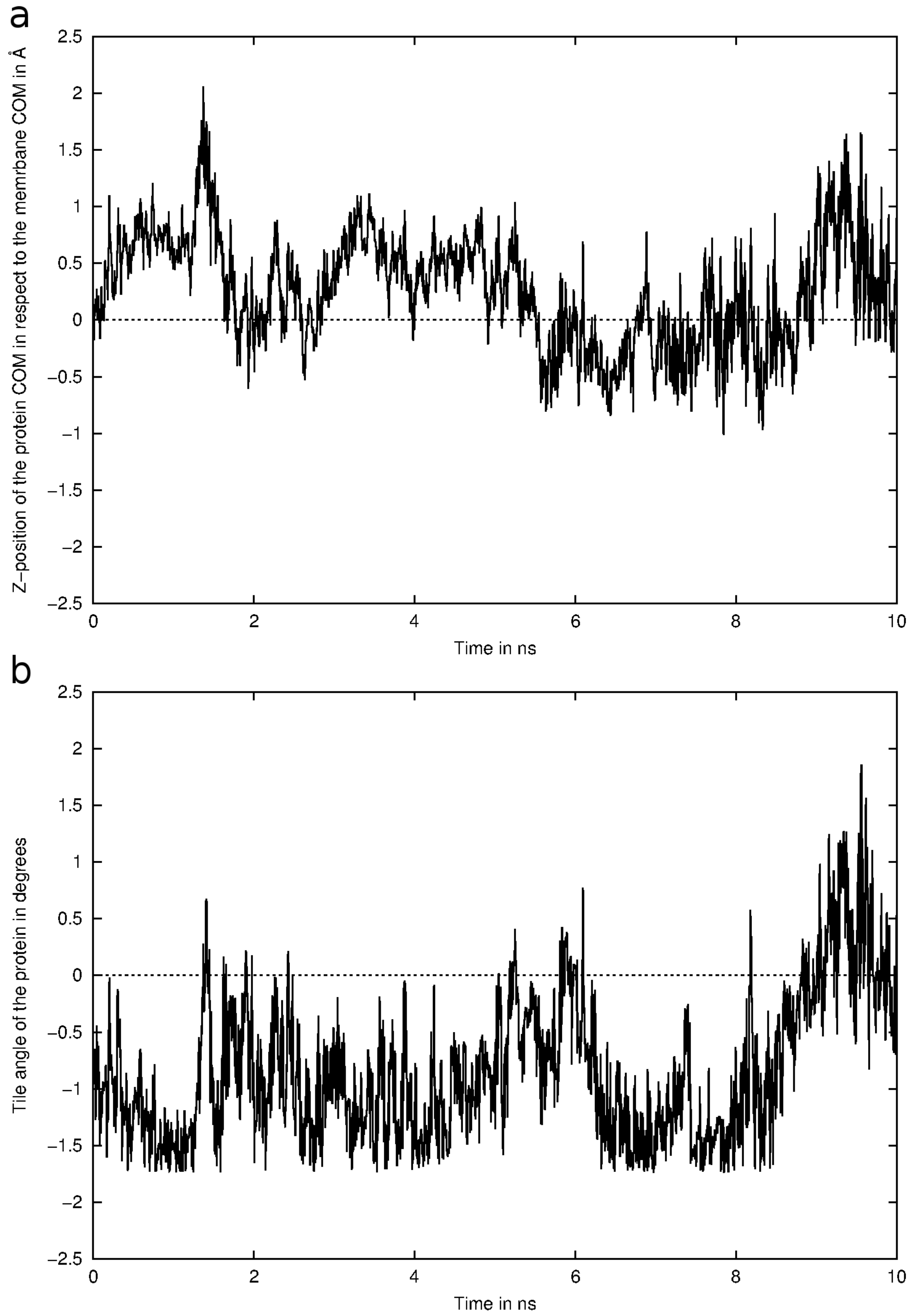

Supplementary Figure S3: Protein fluctuation around the initial position with regard to the POPC membrane. The top panel shows the protein translation along the Z-axis in respect to the membrane during the MD simulation (A) and the bottom panel shows the protein tilt angle in respect to the membrane plane during the MD simulation (B).

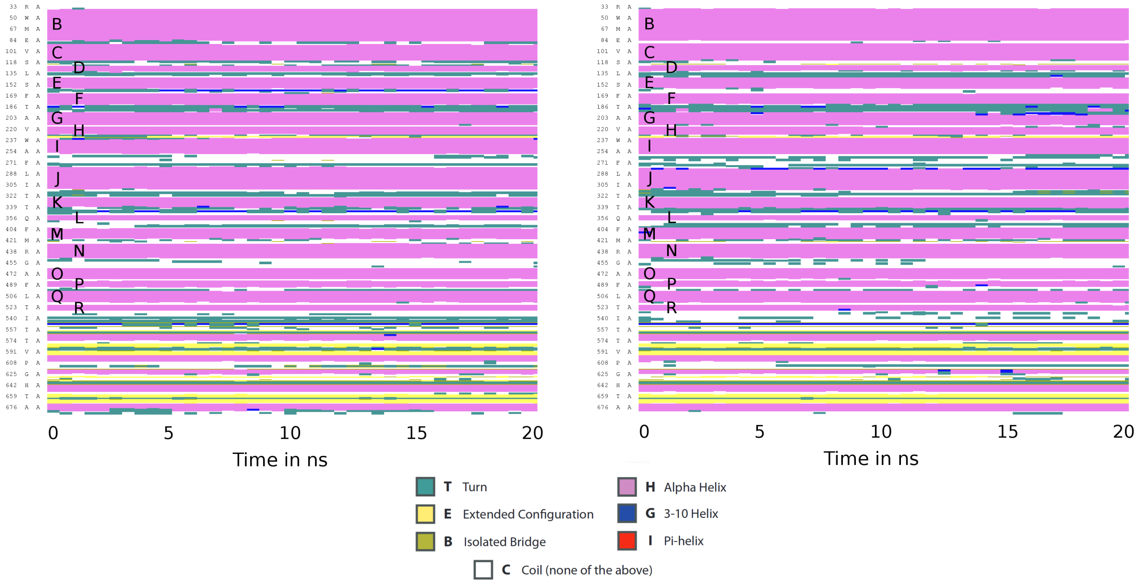

Supplementary Figure S4: Secondary structure variation for every amino acid along the 20 ns MD simulation. The left panel represents the first protomer whereas the second panel represents the second protomer. Letters from B to R represent the TM helices.

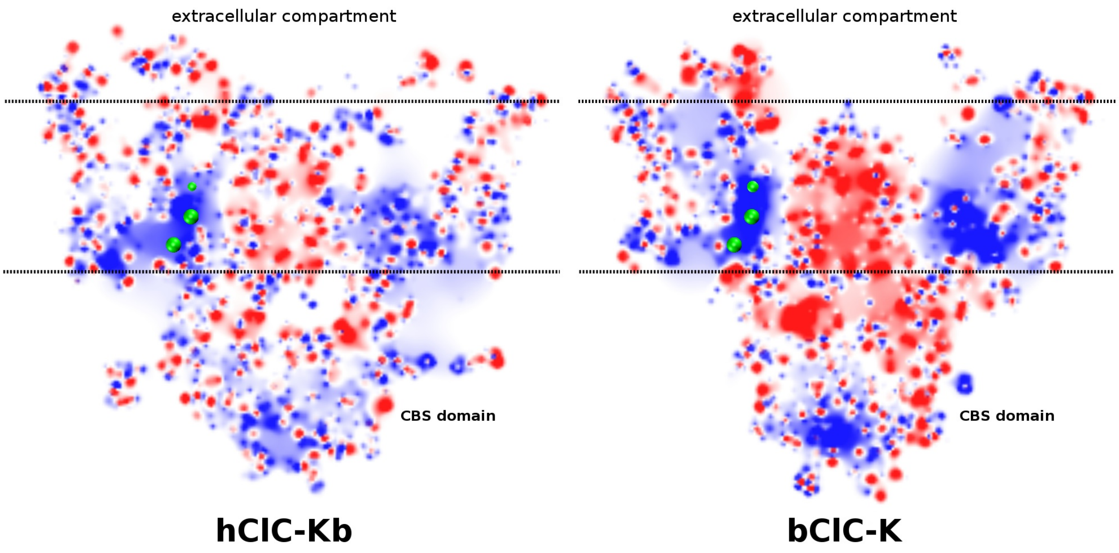

Supplementary Figure S5: Electrostatic potential of the human ClC-Kb and the bovine ClC-K. The electrostatic potentials, coloured from -5 (red) to +5 (blue) kcal.mol^{-1} are mapped on a plane passing by the channel pore. The membrane is represented as dashed lines and the known X-ray positions of the chloride ions are shown as green spheres.

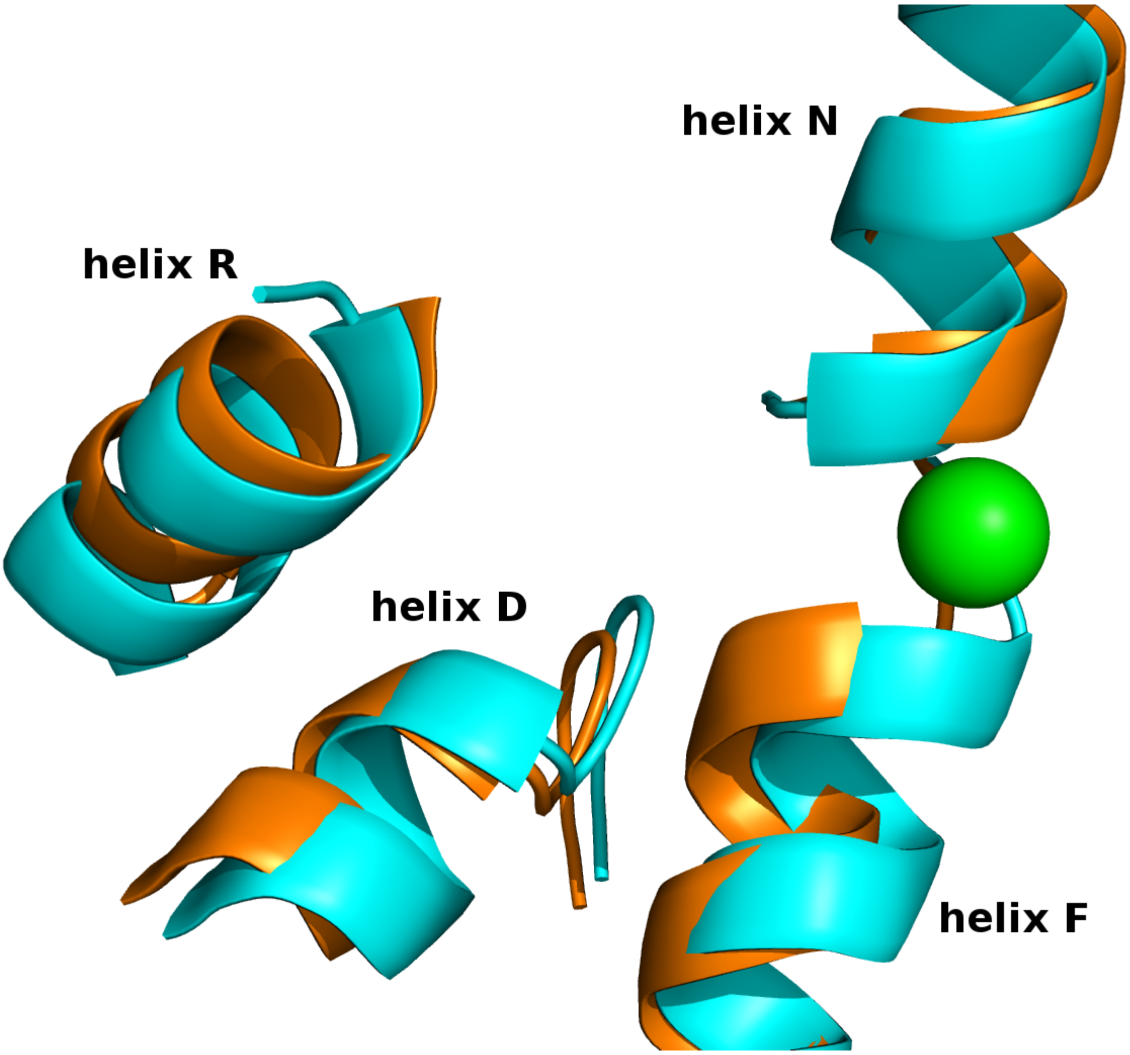

Supplementary Figure S6: Representation of the helix tilt in the ClC channels. The structures of CmCLC (PDB id: 3ORG) and EcClC (PDB id: 1OTS) are shown in a cartoon representation in orange and cyan respectively. The external chloride ion position known from X-ray data (S_{ext}) is shown as a green sphere.

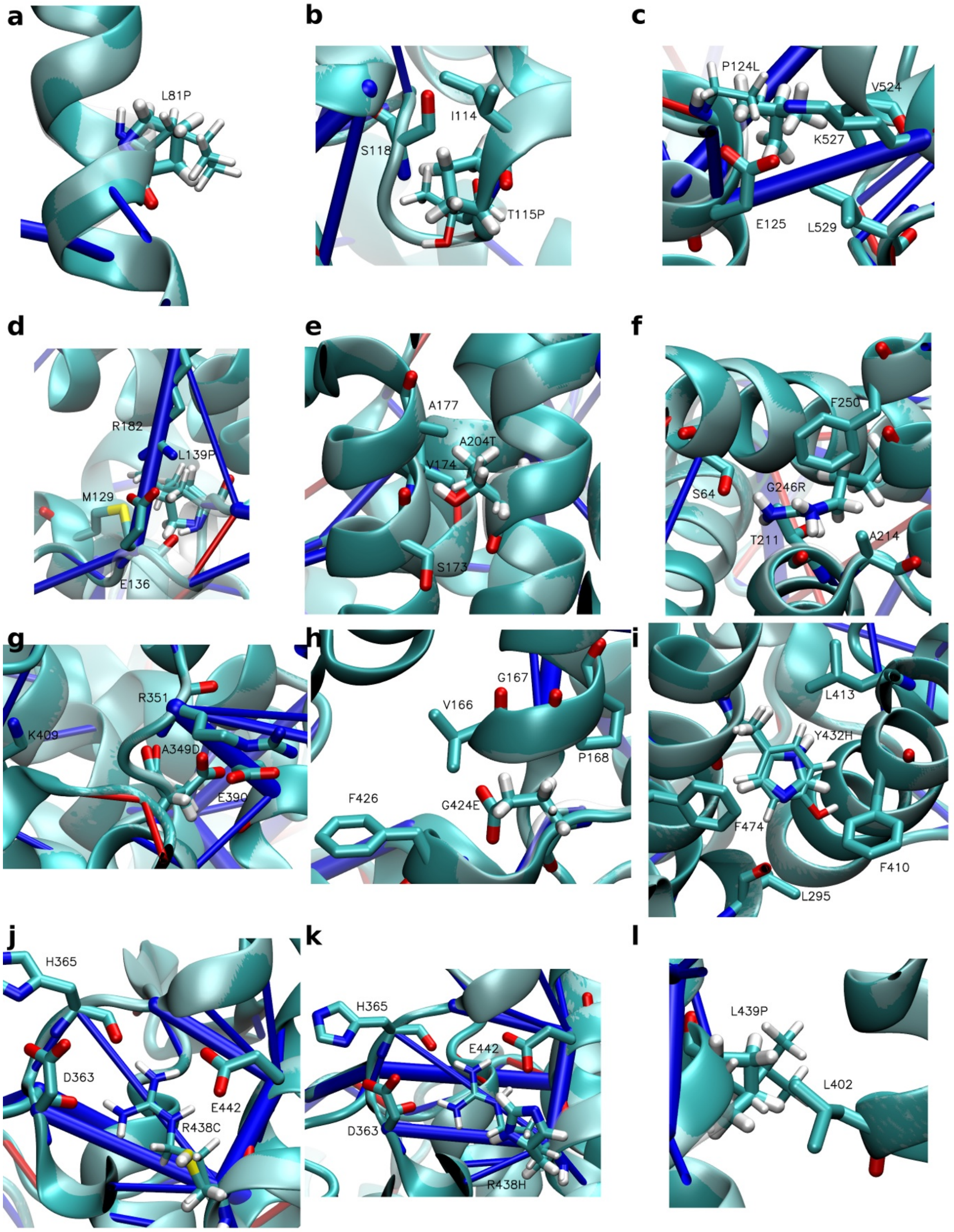

Supplementary Figure S7: Mutant (cartoon cyan) and wild-type (cartoon transparent white) conformation of the ClC-Kb channel. Wild-type side chains are represented as thin sticks and mutant side-chains, as well as surrounding residues, are represented as thick sticks. Red and

blue lines represent interaction energies between the two linked amino acids, the thicker the bars the stronger the interactions. Blue bars represent attractions and red repulsions, the bigger the bars, stronger the interactions. Labels have been added on some interesting residues. Only mutants whose expression and/or surface expression were tested experimentally were plotted: L81P (a), T115P (b), P124L (c), L139P (d), A204T (e), G246R (f), A349D (g), G424E (h), Y432H (i), R438C (j), R438H (k) and L439P (l).

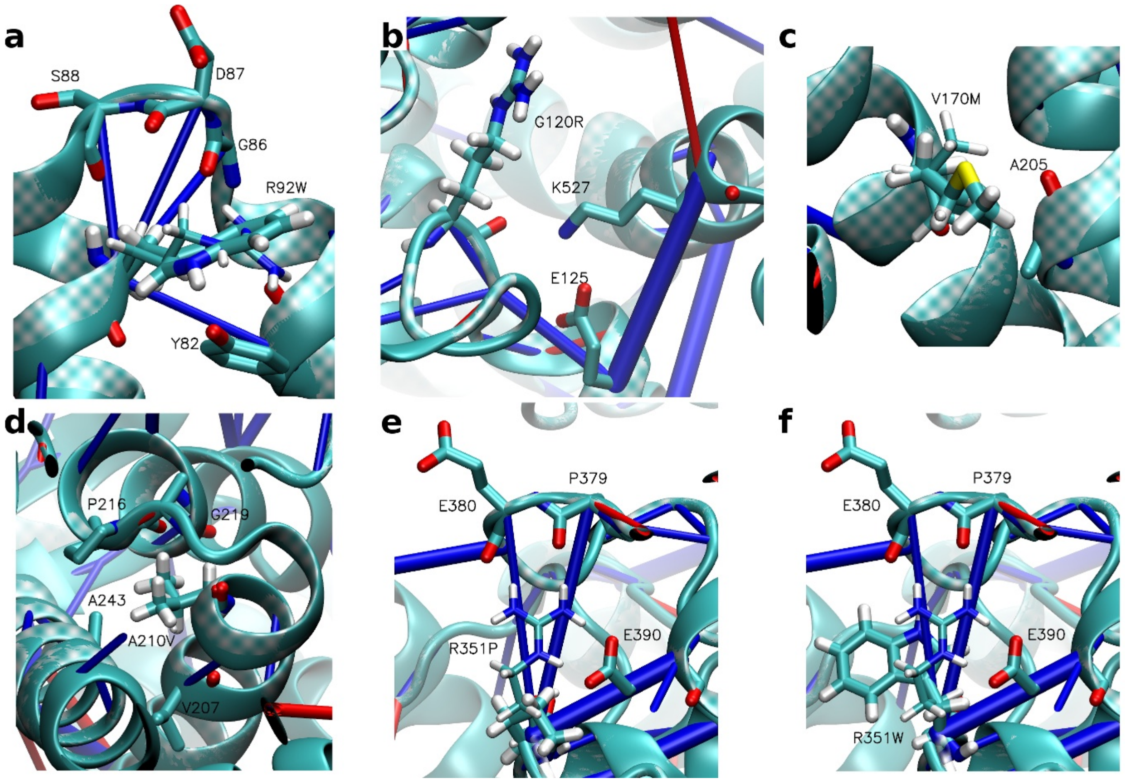

Supplementary Figure S8: Mutant (cartoon cyan) and wild-type (cartoon transparent white) conformation of the ClC-Kb channel. Wild-type side chains are shown in a thin stick representation and mutant side-chains, as well as surrounding residues, are represented as thick sticks. Red and blue lines represent interaction energies between the two linked amino acids, thicker the bars stronger the interactions. Blue bars represented attractions and red repulsions, the bigger the bars, the stronger the interactions. Labels have been added to some residues for orientation. Only mutants with some available experimental data are plotted: R92W (a) G120R (b), V170M (c), A210V (d), R351P (e) and R351W (f).

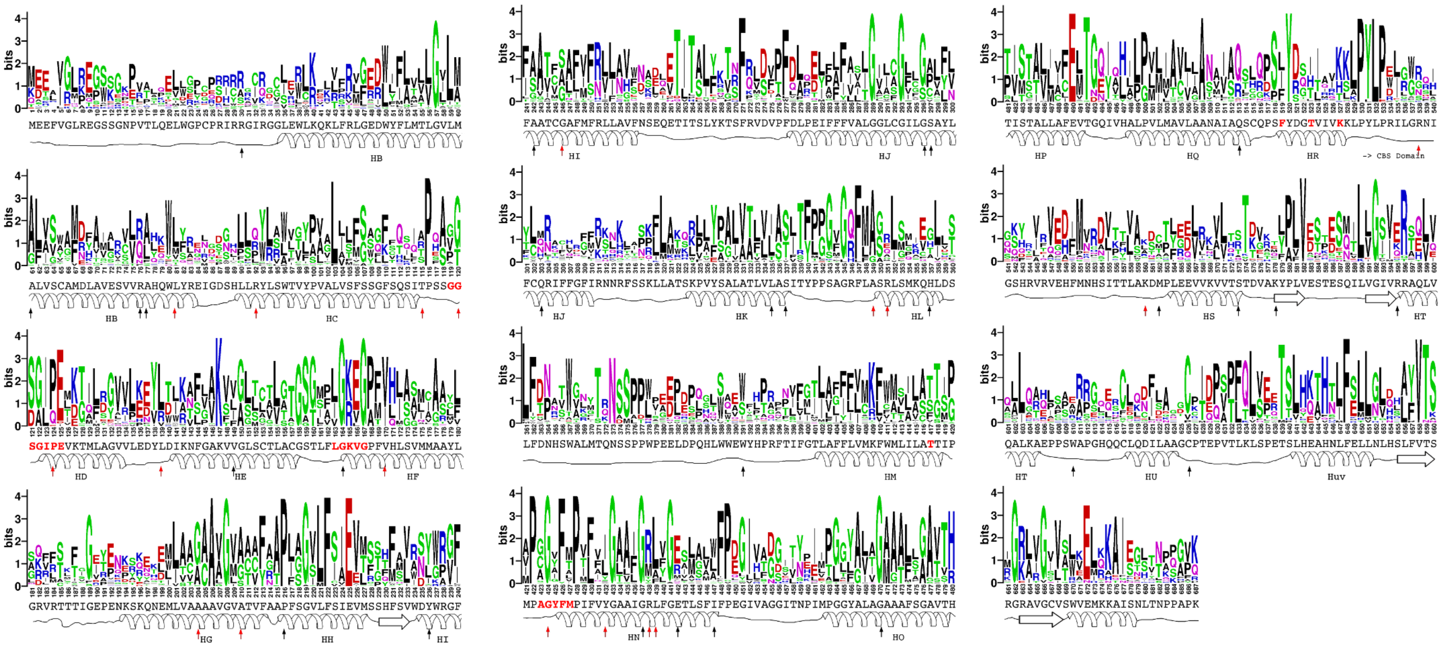

Supplementary Figure S9: WebLogo (http://weblogo.threeplusone.com/) obtained from the multiple alignment of the human ClC-Kb channel with its most homologue sequences. ClC-Kb sequence has been specified under the logo as well as secondary structure elements. The region not modelled in this study (residues 1 to 48) is depicted as a loop. Secondary structures have been named according to the standard nomenclature. Arrows correspond to known amino acid variations, red arrows correspond to mutants where experimental data were available in contrary to black arrows.

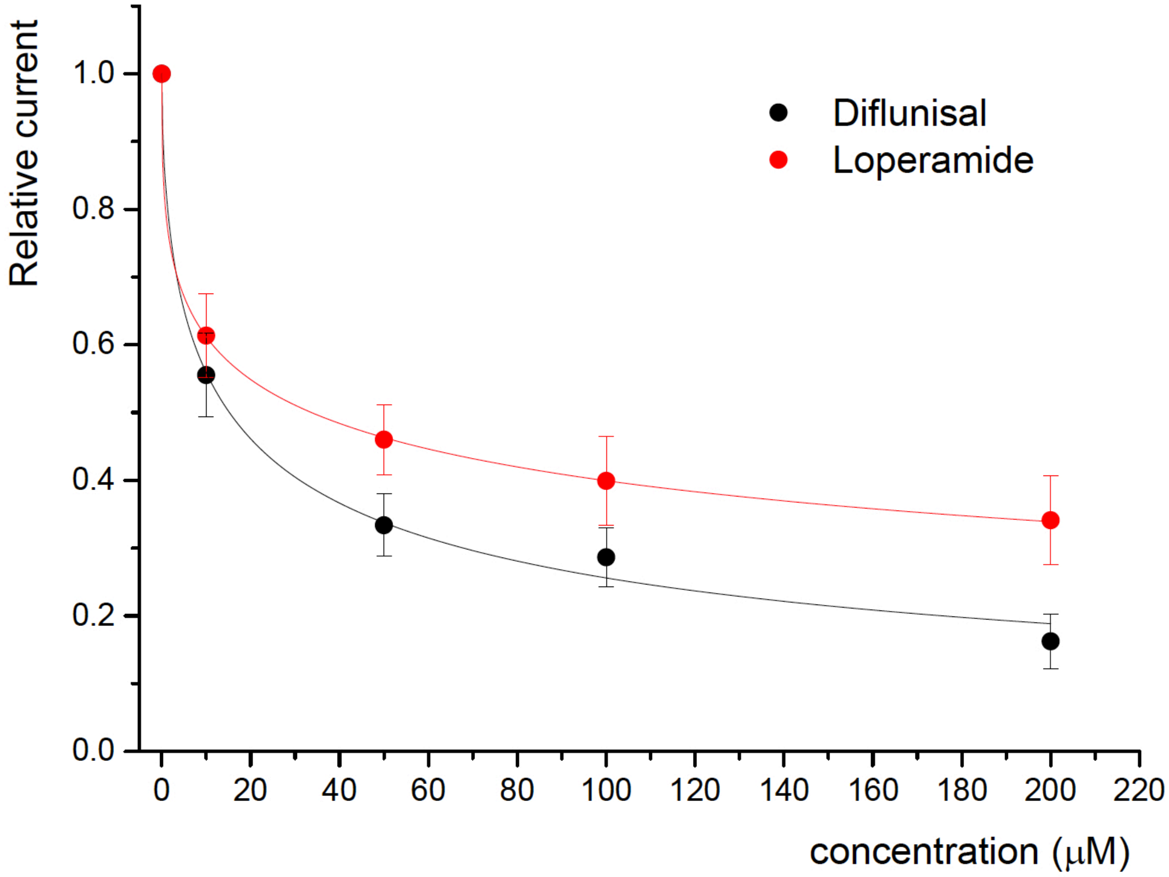

Supplement Figure S10: Dose–response relationships of the ClC-Kb inhibition by diflunisal and loperamide. The relative currents in the presence and the absence of the drugs were plotted versus concentration. Each data point is the mean value of N measurements ± SE. Lines are drawn according to the equation 1/(1+ (x/K)n). Fitting the data gave apparent Kd values of 15.2 and 33.7 μM for diflunisal (N = 5) and loperamide (N = 7), respectively.
